# Microbiome Restructuring: Dominant Coral Bacterium *Endozoicomonas* Species Respond Differentially to Environmental Changes

**DOI:** 10.1128/msystems.00359-22

**Published:** 2022-06-15

**Authors:** Kshitij Tandon, Yu-Jing Chiou, Sheng-Ping Yu, Hernyi Justin Hsieh, Chih-Ying Lu, Ming-Tsung Hsu, Pei-Wen Chiang, Hsing-Ju Chen, Naohisa Wada, Sen-Lin Tang

**Affiliations:** a Biodiversity Research Center, Academia Sinica, Taipei, Taiwan; b Penghu Marine Biology Research Center, Fisheries Research Institute, Council of Agriculture, Penghu, Taiwan; c Molecular and Biological Agricultural Sciences Program, Taiwan International Graduate Program, National Chung Hsing University and Academia Sinica, Taipei, Taiwan; d Graduate Institute of Biotechnology, National Chung Hsing University, Taichung, Taiwan; Michigan State University

**Keywords:** microbiome restructuring, coral-associated bacteria, *Endozoicomonas*, reciprocal transplant, ROS scavenging

## Abstract

Bacteria in the coral microbiome play a crucial role in determining coral health and fitness, and the coral host often restructures its microbiome composition in response to external factors. An important but often neglected factor determining this microbiome restructuring is the ability of microbiome members to respond to changes in the environment. To address this issue, we examined how the microbiome structure of *Acropora muricata* corals changed over 9 months following a reciprocal transplant experiment. Using a combination of metabarcoding, genomics, and comparative genomics approaches, we found that coral colonies separated by a small distance harbored different dominant *Endozoicomonas*-related phylotypes belonging to two different species, including a novel species, “*Candidatus* Endozoicomonas penghunesis” 4G, whose chromosome-level (complete) genome was also sequenced in this study. Furthermore, the two dominant *Endozoicomonas* species had different potentials to scavenge reactive oxygen species, suggesting potential differences in responding to the environment. Differential capabilities of dominant members of the microbiome to respond to environmental change can (i) provide distinct advantages or disadvantages to coral hosts when subjected to changing environmental conditions and (ii) have positive or negative implications for future reefs.

**IMPORTANCE** The coral microbiome has been known to play a crucial role in host health. In recent years, we have known that the coral microbiome changes in response to external stressors and that coral hosts structure their microbiome in a host-specific manner. However, an important internal factor, the ability of microbiome members to respond to change, has been often neglected. In this study, we combine metabarcoding, culturing, and genomics to delineate the differential ability of two dominant *Endozoicomonas* species, including a novel “*Ca.* Endozoicomonas penghunesis” 4G, to respond to change in the environment following a reciprocal transplant experiment.

## INTRODUCTION

Bacteria are among the main microbial partners in the coral holobiont (1). They may play a role in coral health, disease, and nutrient supply (2, 3). A coral colony often accommodates several hundred, if not thousands, of bacterial phylotypes (1, 4), with different bacterial communities residing in coral compartments, such as the coral mucus (5–9), tissue (5–7, 9–11), gastrovascular cavity (12), and skeleton (13–15). These bacterial communities are often diverse, dynamic, and, according to many studies, profoundly influenced by factors such as host specificity and spatiotemporal changes in the surrounding environment (10, 16–20).

Of the factors involved in restructuring the coral-associated bacterial community, environmental changes and host specificity are two major drivers influencing the composition of the bacterial community in corals. In terms of environmental changes, numerous studies have reported shifts in the bacterial community composition of corals in response to variations in temperature (21–25), nutrient load (22, 26), exposure to pathogens (27), and anthropogenic factors (22, 28). Regarding host specificity, the same coral species living in habitats hundreds to thousands of kilometers apart were found to accommodate similar bacterial community profiles (1, 29), whereas adjacent coral colonies of different species had distinct microbiomes (1, 30). Interestingly, several studies have asserted that changes to the coral microbiome composition in response to new environments are host specific; this was tested via transplantation experiments and suggests that microbiome alteration is an acclimatization strategy (23, 31). This microbiome alteration potential is known to vary depending on the host species. For example, Ziegler and coworkers (31) studied variation in the microbiomes of the corals *Acropora hemprichii* and *Pocillopora verrucosa* in a long-term cross-transplantation experiment and identified that *A. hemprichii* harbors a highly flexible microbiome, whereas *P. verrucosa* has a remarkably stable microbiome, even after exposure to different levels of chronic pollution, suggesting that the bacterial communities of different coral species exhibit differential dynamics under environmental change/perturbation.

In most of the studies conducted to date, factors influencing the changes in the coral-associated bacterial community are often external, such as those mentioned above. Only recently have internal factors like host genotype been shown to also influence the coral microbiome (32–34). However, the adaptation capability of bacteria, one hidden but the crucial internal factor, has long been neglected. Theoretically, based on the nature of genetic variations among bacteria, some bacterial phylotypes of the same bacterial group in a community may perform better than others under specific environmental conditions due to higher adaptation capabilities. Therefore, those bacterial phylotypes with higher adaptation capacities could maintain a more stable abundance profile during specific environmental changes and potentially play important functional roles. In other words, along with host selection and environmental influence, changes in the bacterial community may be greatly affected by the capabilities of individual bacterial groups to respond to changes in local conditions. However, this aspect of microbiome restructuring is mostly unexplored.

Whether bacterial groups have different capabilities to respond to changes in local conditions, we first needed to identify a dominant bacterial group often identified in corals with multiple operational taxonomic units (OTUs) or, more recently, amplicon sequence variants (ASVs) from metabarcoding surveys. One such group belongs to the genus *Endozoicomonas* (phylum *Proteobacteria*, class *Gammaproteobacteria*, order *Oceanospirillales*, family *Endozoicomonadaceae* [also *Hahellaceae*]), which, along with many other coral-associated bacteria (35, 36), plays a role in coral health and nutrition regulation (3, 36, 37). A recent study identified that the abundance profiles of certain *Endozoicomonas* OTUs shifted within 12 h under thermal stress (24). If *Endozoicomonas* phylotypes do have a differential capability to respond to change in local conditions, then we can hypothesize that different *Endozoicomonas* phylotypes behave differently and some may remain more stable and colonize longer than others when corals are subjected to environmental change. Another prerequisite to testing the differential capability of bacterial phylotypes is finding a region with differential environmental conditions within a small distance such that geographical variation does not influence the coral microbiome.

**FIG 1:**
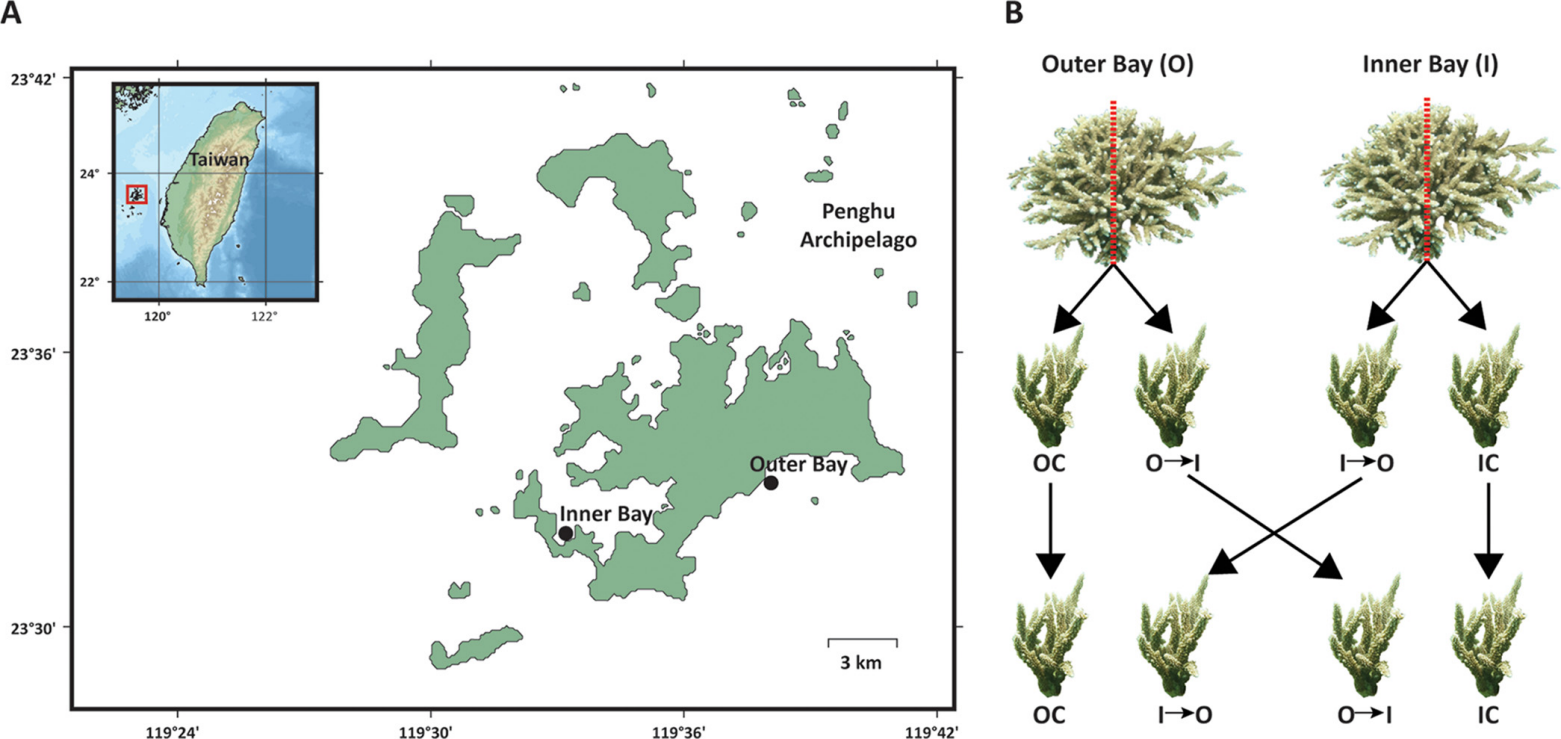
Sampling location and reciprocal transplant experiment overview. (A) Map of the Penghu Archipelago, Taiwan, with two sampling sites: Inner Bay and Outer Bay. (B) Schematic representation of the reciprocal transplant experiment setup with sample codes. OC, Outer Bay Control. IC, Inner Bay Control. O→I, Outer Bay colonies transplanted into the Inner Bay. I→O, Inner Bay colonies transplanted into the Outer Bay.

The Penghu Archipelago, located in the Taiwan Strait (Fig. 1A), has been proposed to be a climate change refugium for corals and has a unique thermal regime, governed by the warm Kuroshio Current in the summer and cold China Coastal Current in the winter (24, 38). These factors make it an ideal location for an experimental site. The semiclosed Chinwan Inner Bay (here Inner Bay) of Penghu has suffered substantial marine biodiversity losses, including significant damage to marine aquaculture, wild fisheries, and coral bleaching due to extreme weather events in the winter (39). Furthermore, based on regional news and government reports, domestic sewage dumping, the presence of a shipping port, and aquaculture practices have increased the concentrations of nitrogen and ammonia in the calmer waters of the Inner Bay compared to the Outer Bay region, threatening corals. The contrasting local environmental conditions between the Inner and Outer Bays make them excellent sites to study the response of locally acclimated dominant coral microbial communities, e.g., *Endozoicomonas*, as members of this genus show lower relative abundance in degraded or anthropogenically impacted reefs compared to the one that is healthy (28). Further, contrasting conditions in the sites also help trace coral microbiome restructuring at a fine scale and test the differential capability of dominant coral-associated bacteria to respond to environmental change.

We examined how the microbial community restructures after reciprocal transplant and whether the *Endozoicomonas* phylotypes have differential responses to change in local conditions using colonies of the coral *Acropora muricata* (genus *Acropora*) in the Penghu Archipelago, Taiwan. These coral species in the Penghu Archipelago have been reported to harbor *Endozoicomonas* as their dominant bacteria (24). We conducted a longitudinal (9-month) *in situ* reciprocal transplant experiment with repeated sampling, where coral colonies from the semiclosed Inner Bay were transplanted into the open ocean region of the Outer Bay and vice versa. Furthermore, we aimed to isolate, culture, and characterize dominant *Endozoicomonas* members to provide genomic insights into how bacteria adapt to these environments.

## RESULTS

### Sampling and sequencing overview.

**FIG 2:**
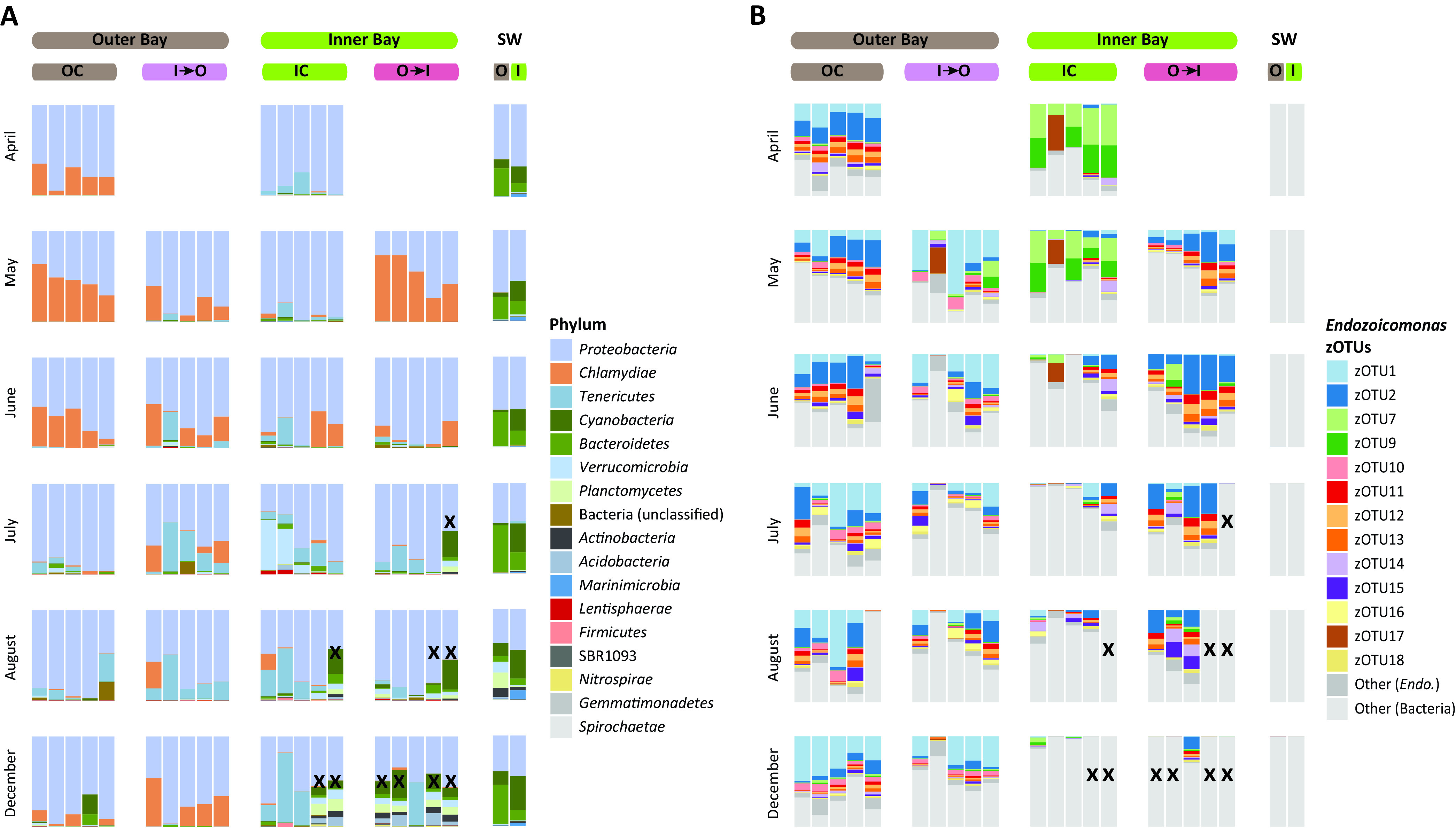
Bacterial community composition overview. (A) Relative abundance-based bacterial community composition at the phylum level across all sample sets (IC, OC, I→O, and O→I). (B) Relative abundance of different *Endozoicomonas* zOTUs across all sample sets. X denotes dead colonies.

We collected a total of 110 coral and 12 seawater samples from the experiment, of which 10 coral fragments were removed (all from the Inner Bay) during the experiment (marked with an X in Fig. 2A and B), as they appeared to be dead. These dead samples were only used to help contrast with the microbial community compositions of healthy corals and were later removed before downstream analyses, including α and β diversity analyses. At the end of the experiment, we had 100 coral and 12 seawater samples. A total of 2,015,935 high-quality reads (an average of 16,524 reads per sample) were obtained after removing chimeras and poor-quality reads from the 110 coral and 12 seawater samples. These reads were denoised into 2,064 zero-radius operational taxonomic units (zOTUs). Healthy corals (*n *= 100) had 1,815,002 reads (range, 6,092 to 67,598) and 1,966 zOTUs, and seawater (*n* = 12) had 149,638 reads (range, 6,897 to 19,777) and 1,521 zOTUs.

### Coral and seawater microbiomes differ in bacterial diversity and compositions.

The coral and seawater samples were significantly different in bacterial diversity and evenness, measured through zOTU richness (see Fig. S1A in the supplemental material), Shannon (Fig. S1B) and Chao1 (Fig. S1C) diversities, and inverse Simpson evenness (Fig. S1D). The seawater samples had more than twice the zOTU richness and Shannon and Chao1 diversities of the coral samples. August samples showed an unusual alpha diversity pattern, particularly in seawater samples; this could be because the samples were collected during heavy rainfall (±3 days). We observed an increase in richness and Chao1 between O→I and OC samples, but no apparent differences were observed between I→O and IC samples. In terms of corals at different locations, there was no significant difference in calculated alpha diversity measures between control and transplant samples from the Inner and Outer Bays (Fig. S1A to D).

10.1128/msystems.00359-22.1 FIG S1 Alpha diversity analysis. (A) Richness. (B) Shannon-Weiner index. (C) Chao1. (D) Inverse Simpson. No significant differences were observed in coral samples from the Inner or Outer Bay (IC, OC, I→O, and O→I). Alpha diversity estimates were significantly (*P* < 0.05) different between coral and seawater samples as denoted by different group annotations (a and b). Download FIG S1, PDF file, 1.6 MB. Copyright © 2022 Tandon et al. 2022 Tandon et al. https://creativecommons.org/licenses/by/4.0/ This content is distributed under the terms of the Creative Commons Attribution 4.0 International license.

*Proteobacteria* (specifically class *Alphaproteobacteria*) (average relative abundance, 51.71%) and *Bacteroidetes* (22.76%) (class *Flavobacteriia*) were the dominant phyla in seawater samples across all time points, followed by *Cyanobacteria* (17.75%), which was particularly abundant in Inner Bay samples. There was heavy rainfall during the week in August that samples were collected, and we noticed a higher abundance of *Marinimicrobia* (class *Marinimicrobia* SAR406 clade), *Planctomycetes*, and *Verrucomicrobia* in those samples, which might explain the different bacterial diversity and evenness results obtained that month. On the contrary, coral samples were dominated by *Proteobacteria* (specifically class *Gammaproteobacteria*) (73.08%), *Chlamydiae* (13.70%), and *Tenericutes* (8.99%) (class *Mollicutes*). Dead coral samples had a bacterial community composition similar to that of seawater samples (Fig. 2A and Fig. S2).

10.1128/msystems.00359-22.2 FIG S2 Bacterial community composition of coral and seawater samples at the class taxonomic rank. Download FIG S2, PDF file, 0.5 MB. Copyright © 2022 Tandon et al. 2022 Tandon et al. https://creativecommons.org/licenses/by/4.0/ This content is distributed under the terms of the Creative Commons Attribution 4.0 International license.

### Changes in the coral microbial community throughout the reciprocal transplant.

*Proteobacteria* was the dominant phylum across all sample groups (control, IC and OC; transplant, I→O and O→I) from the two locations throughout the experiment. We observed shifts in the microbial community of control samples (IC and OC) from April to August and December. The relative abundance of *Chlamydiae* (family *Simkaniaceae*), the second abundant phylum in the Outer Bay (OC), with all zOTUs (10 in count) belonging to “Unclassified Simakaniaceae,” decreased from April to August before increasing slightly in December. *Tenericutes* (specifically *Mollicutes*), the second dominant phylum in the Inner Bay control samples (IC), increased in abundance over time, peaking in December. One zOTU annotated as “Unclassified Entomoplasmatales” had the highest abundance among different members of *Tenericutes*, including zOTUs belonging to *Acholeplasma*, *Mycoplasma*, “*Candidatus* Bacilloplasma,” and “*Candidatus* Hepatoplasma.” In July, we observed a sudden spike in *Verrucomicrobia* abundance in two samples from IC, but soon after in August the community composition became similar to that in June. We also observed patterns of community dynamics in *Chlamydiae* and *Tenericutes* in cross-transplant samples (I→O and O→I) over the sampling period. *Chlamydiae* became the most dominant group in O→I (May) samples, but its abundance decreased sharply thereafter, whereas in I→O samples, *Chlamydiae* and *Tenericutes* both remained stable, with *Chlamydiae* being dominant in May and June and *Tenericutes* being dominant in July and August (Fig. 2A and Fig. S2).

At the genus taxonomic rank, *Endozoicomonas* species were the most dominant. Forty-eight zOTUs (out of 2,064) were taxonomically classified as *Endozoicomonas*. These 48 zOTUs accounted for an average of ~54% relative abundance in coral fragments and 0% in sea water samples. Of these 48 zOTUs, 13 contributed ~90% of the total *Endozoicomonas* abundance. Interestingly, IC and OC samples harbored different dominant *Endozoicomonas* zOTUs, with zOTU1 and zOTU2 being dominant in OC and zOTU7 and zOTU9 in IC. Across the sampling time, we also observed shifts in the dominant *Endozoicomonas* phylotypes. zOTU2 was dominant from April to June, whereas zOTU1 became dominant in July-December OC samples. In IC samples, zOTU7 was dominant from April to May, but after that its relative abundance declined (Fig. 2B). It is also worth noting that a significant decline in the *Endozoicomonas* abundance was observed in IC samples in July, August, and December (Fig. 2B), suggestive of location dependence.

In cross-transplant samples, the *Endozoicomonas* phylotypes from OC remained resistant to change when transplanted in the Inner Bay (O→I), with zOTU2 being dominant across all sampling times. For I→O transplanted samples, however, instead of *Endozoicomonas* phylotypes from IC, we observed that zOTU1, another dominant *Endozoicomonas* phylotype in OC samples, was dominant (Fig. 2B), suggesting that the phylotypes had different robustness under different environmental scale disturbances.

### Location-dependent robustness in the coral microbiome.

**FIG 3:**
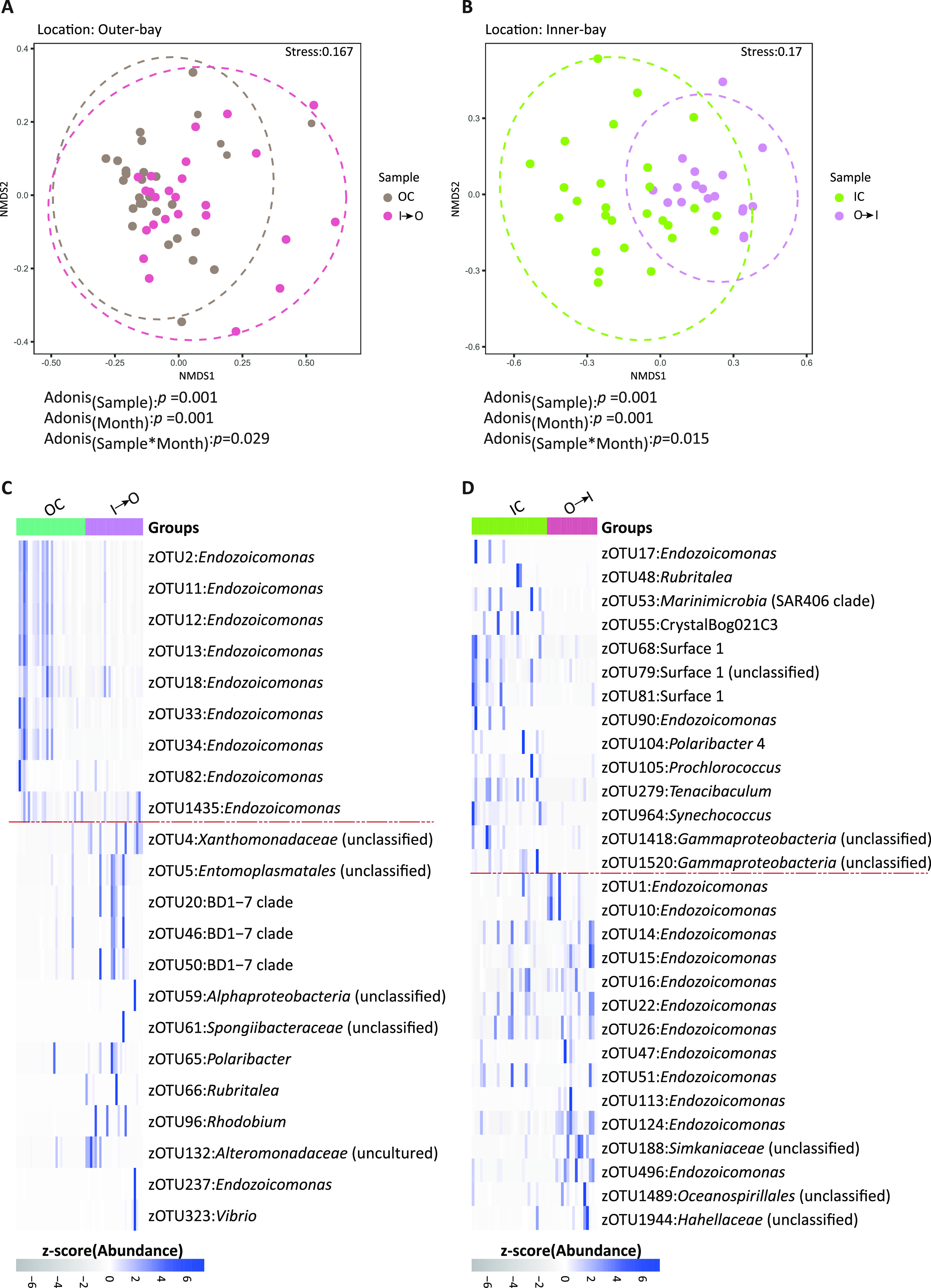
Location-dependent bacterial community structure and differentially abundant bacterial community. Plots based on nonmetric multidimensional scaling (nMDS) of Bray-Curtis dissimilarity of bacterial community composition at the zOTU level associated with different locations. Final locations were Outer Bay (OC and I→O) (A) and Inner Bay (IC and O→I) (B). PERMANOVA identified sample location, month, and interaction terms that are significant factors in determining the *Acropora muricata* microbiome. LEfSe result-based differentially abundant zOTUs over sample groups in the Outer Bay (OC and I→O) (C) and Inner Bay (IC and O→I) (D). zOTUs above the dotted red lines are differentially abundant in control (OC and IC), and the ones below are differentially abundant in transplant samples (I→O and O→I).

The dispersion of homogeneity analysis identified that the bacterial community in corals with the Inner Bay as the final location (IC and O→I) were significantly different from each other (analysis of variance [ANOVA], *F* = 9.23, *P* < 0.001), whereas samples whose final destination was the Outer Bay (OC and I→O) had no significant difference (ANOVA, *F* = 1.98, *P* > 0.05), indicating that the microbiome had location specificity. Therefore, samples whose final destinations were the Inner Bay and Outer Bay were analyzed independently to test for differences in community composition between the control and transplant groups. Ordination analysis using nonmultidimensional scaling (nMDS), followed by permutational multivariate analysis of variance (PERMANOVA), identified the significant influence of coral sample, sampling month, and their combined effect (interaction term) (Fig. 3A). Ellipses with a 95% confidence interval suggested that samples for which the Outer Bay was their final location (OC and I→O) were more similar to each other than to samples for which the Inner Bay was their final location (IC and O→I). These findings support locational variability and differential robustness in the coral microbiome (Fig. 3A and B). Transplanted samples (O→I) clustered tightly compared to IC samples, indicating less variability after transplantation in the transplant samples. However, highly overlapping ellipses were observed for OC and I→O samples, suggesting a highly similar microbial community in the control (OC) and transplanted samples from the Inner Bay (I→O) (Fig. 3A and B).

### Differentially abundant microbiome dominated by *Endozoicomonas*-related phylotypes.

Linear discriminant analysis effect size (LEfSe) analysis identified differentially abundant zOTUs of different taxa across all the sampling groups: nine zOTUs were differentially abundant in OC samples, 13 in I→O samples, and 16 in IC and O→I samples (Fig. 3C and D). Interestingly, all the differentially abundant zOTUs in the OC samples belonged to *Endozoicomonas*, but zOTUs belonging to diverse taxa, including the BD1-7 clade (*Gammaproteobacteria*), *Entomoplasmatales* (phylum *Tenericutes*; class *Mollicutes*), and *Alteromonadaceae* (class *Gammaproteobacteria*), were differentially abundant in I→O samples (Fig. 3C). Similarly, out of the 16 zOTUs that were differentially abundant in O→I samples, 13 were *Endozoicomonas*; IC samples also had zOTUs belonging to diverse taxa that were differentially abundant, including Surface 1_ge (class *Alphaproteobacteria*), *Synechococcus* (class *Cyanobacteria*), and others (Fig. 3D).

### Phylogenetic analysis of dominant *Endozoicomonas* zOTUs and a novel cultured species.

**FIG 4:**
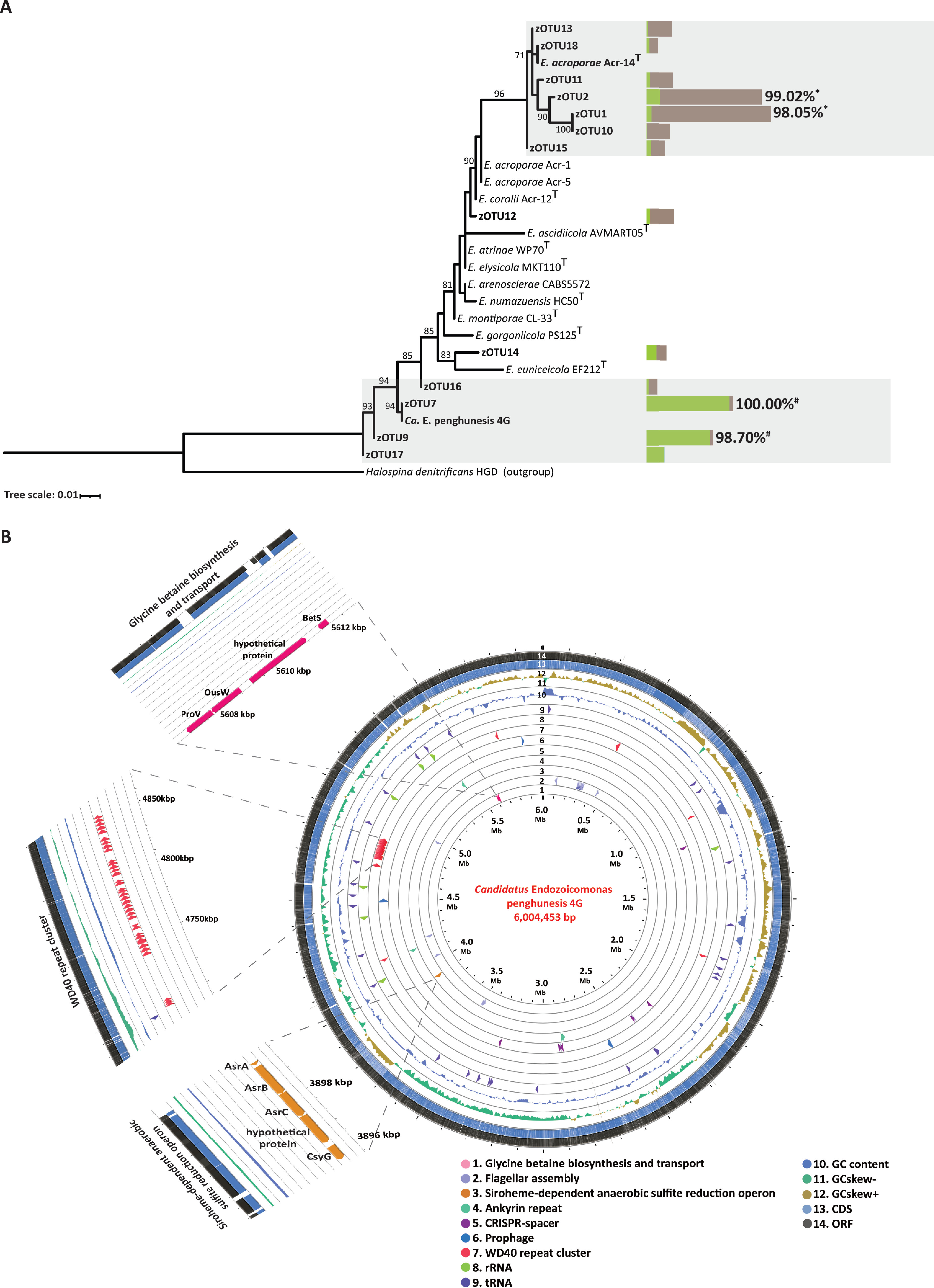
Phylogenetic tree and genome map of “*Candidatus* Endozoicomonas penghunesis” 4G. (A) Phylogenetic tree of dominant zOTUs and 16S rRNA sequences of “*Ca.* Endozoicomonas penghunesis” (Copy1) and Endozoicomonas acroporae Acr-14^T^. Horizontal bars denote the relative abundance of selective zOTUs in the Inner (green) and Outer (brown) Bays. The percent values denote the percent identity between the zOTU and cultured 16S rRNA copy. #, “*Ca.* Endozoicomonas penghunesis”; *, *E. acroporae* Acr-14^T^. Shaded regions are considered to belong to one bacterial species. (B) Whole-genome map of “*Ca.* Endozoicomonas penghunesis” 4G drawn in CGViewer with concentric circles depicting distinct features. The map also highlights the concentration of WD40 domain proteins, Siroheme-dependent anaerobic sulfite reduction operon and glycine-betaine biosynthesis and transport pathways.

The high abundance of *Endozoicomonas*-related phylotypes in the coral samples and their differential robustness after transplantation (i) motivated us to determine their phylogenetic position and (ii) provided an opportunity to isolate and culture these phylotypes. A phylogenetic tree based on 16S rRNA gene sequences and the percent identity match between these sequences confirmed that zOTU1 and zOTU2 were 99.02 and 98.05% identical (16S rRNA V6-V8 region), respectively, to Endozoicomonas acroporae Acr-14^T^ (Fig. 4A). They also formed a distinct clade with zOTU10, zOTU13, zOTU15, and zOTU18 (Fig. 4A). These zOTUs (zOTU10, -13, -15, and -18) were also >97% identical to *E. acroporae* Acr-14^T^ 16S rRNA gene (Fig. S3A). However, zOTU7, zOTU9, zOTU16, and zOTU17 formed a separate clade away from any cultured *Endozoicomonas* species (Fig. 4A). zOTU7 was 100% identical to a newly isolated and cultured species (“*Ca.* Endozoicomonas penghunesis” 4G) described in this study (described below), and zOTU9 had 98.70% identity (16S rRNA gene V6-V8 region) with “*Ca.* Endozoicomonas penghunesis” 4G (Fig. 4A). zOTU17 and zOTU16 were also >97% identical to “*Ca.* Endozoicomonas penghunesis” 4G 16S rRNA gene (Fig. S3B). A genomic analysis of “*Ca.* Endozoicomonas penghunesis” 4G identified seven copies of 16S rRNA (described below) based on percent similarity; 16S rRNA gene copy 1 (Fig. S3C) was used as the representative for the phylogenetic tree in Fig. 4A. We also performed phylogenetic analysis for all copies of 16S rRNA present in “*Ca.* Endozoicomonas penghunesis” and *E. acroporae* Acr-14^T^ (Fig. S3D).

10.1128/msystems.00359-22.3 FIG S3 16S rRNA gene percentage identity heatmaps (A) between *Endozoicomonas*-related zOTUs and *E. acroporae* Acr-14^T^, (B) “*Ca.* Endozoicomonas penghunesis 4G” 16S rRNA (copy1) and zOTUs related to *Endozoicomonas*. (C) “*Ca.* Endozoicomonas penghunesis” 4G 16S rRNA copies percent identity to each other. (D) Phylogenetic tree of 16S rRNA copies of “*Ca.* Endozoicomonas penghunesis 4G” with Endozoicomonas acroporae Acr-14^T^ as an outgroup. zOTUs represented here is the same used in Fig. 4A. (E) Heatmap of average nucleotide identity across genomes from genus *Endozoicomonas*. Download FIG S3, PDF file, 0.6 MB. Copyright © 2022 Tandon et al. 2022 Tandon et al. https://creativecommons.org/licenses/by/4.0/ This content is distributed under the terms of the Creative Commons Attribution 4.0 International license.

### Description of “*Ca.* Endozoicomonas penghunesis” 4G.

“*Ca.* Endozoicomonas penghunesis” 4G is a Gram-negative, facultatively anaerobic, and slightly motile bacterium that forms beige-colored colonies (size, 2.14 by 0.66 μm) and is slightly susceptible to the antibiotics streptomycin and ampicillin. No catalase enzymatic activity was reported for this bacterium, but the bacterial culture was trypsin and oxidase positive (Table S2). This new bacterial species was identified to tolerate the specified temperature (15 to 35°C) and salinity (5 to 30 PSU) ranges (Table S3). The pH range for growth was pH 6.0 to 10.0, with optimal growth observed at a slightly alkaline pH (pH 8.0). Scanning electron microscopy (SEM) and transmission electron microscopy (TEM) analyses identified rod-shaped cells (Fig. S4C) surrounded by a possible mucus lining (Fig. S4A) and with structures that appeared to be granules or vacuoles in the cell (Fig. S4B). A 16S rRNA gene sequence blast search identified the closest cultured relative to be Endozoicomonas montiporae CL-33 (GenBank accession no. CP013251) with 96.17% identity; based on a species identity cutoff of 97%, this suggests that the bacterium is a novel *Endozoicomonas* species. Further, average nucleotide identity (ANI) analysis on genome sequences confirmed our assertion that “*Ca.* Endozoicomonas penghunesis” 4G is a novel species (Fig. S3E).

10.1128/msystems.00359-22.4 FIG S4 Electron micrographs of “*Candidatus* Endozoicomonas penghunesis 4G”. (A) Negative stain by 2% phosphotungstate, (B) thin section, and (C) surface of a bacterial colony. Micrographs in panels A and B were imaged under transmission electron microscope; panel C was observed under cyro-scanning electron microscopy. Download FIG S4, PDF file, 12.4 MB. Copyright © 2022 Tandon et al. 2022 Tandon et al. https://creativecommons.org/licenses/by/4.0/ This content is distributed under the terms of the Creative Commons Attribution 4.0 International license.

10.1128/msystems.00359-22.9 TABLE S2 Differential phenotypic characteristics of “*Candidatus* Endozoicomonas penghunesis 4G” and *E. acroporae* Acr-14^T^. Download Table S2, DOCX file, 0.02 MB. Copyright © 2022 Tandon et al. 2022 Tandon et al. https://creativecommons.org/licenses/by/4.0/ This content is distributed under the terms of the Creative Commons Attribution 4.0 International license.

10.1128/msystems.00359-22.10 TABLE S3 “*Candidatus* Endozoicomonas penghunesis 4G” and related *Endozoicomonas* species strain growth conditions. Download Table S3, DOCX file, 0.02 MB. Copyright © 2022 Tandon et al. 2022 Tandon et al. https://creativecommons.org/licenses/by/4.0/ This content is distributed under the terms of the Creative Commons Attribution 4.0 International license.

### Genome assembly features of “*Ca.* Endozoicomonas penghunesis” 4G.

The genome of “*Ca.* Endozoicomonas penghunesis” 4G was first assembled using Nanopore reads and later polished with quality filtered Illumina reads, resulting in a single contig of 6,004,453 bp and *N*_50_ of 6,004,453. Genome completeness, contamination, and strain heterogeneity were estimated to be 97.52, 0.98, and 0%, respectively. Out of 573 single-copy marker genes (c_Gammaproteobacteria) from the checkM database (40), 493 genes were present only once, five single-copy markers were duplicated, and nine were missing. Based on the criteria of “minimum information for single amplified and metagenome-assembled genome of bacteria” (41), our genome can be considered “finished.” The GC content of the genome was 49.1%, which is similar to that of other *Endozoicomonas* species.

### Genomic features of “*Ca.* Endozoicomonas penghunesis” 4G.

A total of 5,019 genes and 4,913 coding DNA sequences (CDS) were predicted from the genome. We annotated seven copies of 16S, nine of 23S, eight of 5S rRNA genes, and 80 tRNAs. A sequence similarity analysis of 16S rRNA gene copies revealed that all copies were at least 98.76% identical, with four copies >99.28% identical and two 100% identical to each other (Fig. S3C). Copy-1 of the 16S rRNA gene was used as a representative sequence to classify the closest relative of dominant *Endozoicomonas* zOTUs identified in this study (Fig. 4A). There were no CRISPR elements, and only one prophage was identified in the genome. Out of the 5,019 genes predicted, more than 50% (2,721) were annotated to be hypothetical. Since *Endozoicomonas* species have been exclusively isolated from their marine eukaryotic hosts, including “*Ca.* Endozoicomonas penghunesis” 4G, we searched for eukaryote-like proteins (ELPs) and identified 43 WD40 domain proteins (WD40), four ankyrin repeat proteins (ARPs), and 12 tetratricopeptide repeat proteins (TRPs). Almost all the WD40 domain-containing proteins were arranged consecutively (Fig. 4B) and flanked by transposons. Most (27 out of 41) of the WD40 domain proteins were annotated as TolB protein from the Tol-Pal system, which is important for maintaining cellular integrity, and others (14) were classified as hypothetical proteins. A wide array of secretory proteins was also annotated with 248 type III secretion system (T3SS), 50 type IV secretion system (T4SS), and 10 type VI secretion system (T6SS) effectors annotated from the proteome.

### Metabolic repertoire of “*Ca.* Endozoicomonas penghunesis” 4G.

RAST classified only 36% (1,756) of the total genes into subsystems. Subsystems (i) carbohydrates and (ii) cofactors, vitamins, prosthetic groups, and pigments had the highest number of annotated genes, 270 and 238, respectively (Fig. S5). In the stress response subsystem, 108 genes were annotated, most of which were related to oxidative stress response (46 genes), followed by heat shock response (18) and detoxification response (16). Interestingly, within the osmotic stress response, we identified genes for betaine transport via ATP-binding cassette transporter BetS (high-affinity choline uptake protein BetS), arranged in tandem with an l-proline glycine betaine ABC transport system permease (ProV and OusW) (Fig. 4B). Multiple copies of superoxide dismutase, alkyl hydroperoxide reductase, and methionine sulfoxide reductase genes were also identified in the genome.

10.1128/msystems.00359-22.5 FIG S5 RAST annotation. Subsystem level annotation of open reading frames (as percentages) from “*Ca.* Endozoicomonas penghunesis” and *E. acroporae* Acr-14^T^ using the Rapid Annotation Server Tool. Download FIG S5, PDF file, 0.5 MB. Copyright © 2022 Tandon et al. 2022 Tandon et al. https://creativecommons.org/licenses/by/4.0/ This content is distributed under the terms of the Creative Commons Attribution 4.0 International license.

“*Ca.* Endozoicomonas penghunesis” 4G had genes encoding essential amino acids and pathways including glycolysis and tricarboxylic acid cycle; genes for converting nitrate to nitrite (NapAB, KEGG Ontology no. K02567) and ammonia to l-glutamate were identified, but none were related to the conversion of nitrite to ammonia. Assimilatory and dissimilatory sulfate reduction and oxidation pathways were also completely absent. Furthermore, no genes related to the uptake of extracellular taurine or its metabolism to sulfite were identified. Interestingly, siroheme biosynthesis and siroheme-dependent anaerobic sulfite reduction operons were present in “*Ca.* Endozoicomonas penghunesis” 4G (Fig. 4B). We also identified genes arranged in an operon-like manner for anaerobic glycerol degradation.

### Comparing *E. acroporae* and “*Ca.* Endozoicomonas penghunesis” physiological and genomic features.

*Endozoicomonas* phylotypes belonging to *E. acroporae* and “*Ca.* Endozoicomonas penghunesis” 4G were dominant in colonies of coral *Acropora muricata* in the Outer and Inner Bay, respectively (Fig. 2B and 4A). This selective dominance could be attributed to multiple factors, including bacterial physiological and genetic repertoire. Therefore, we compared the two species; “*Ca.* Endozoicomonas penghunesis” had a wider growth temperature range than *E. acroporae* and was slightly motile. *E. acroporae*, on the other hand, was nonmotile (Table S2) and had a wider salinity and growth pH range (Table S3). A wider growth temperature range of “*Ca.* Endozoicomonas penghunesis” and its dominance in the Inner Bay aligns with the high variation of temperature fluctuations (from summer to winter) observed in the calmer waters of the Inner Bay. Comparing the metabolic repertoire of the two species, we found that genes for dimethylsulfoniopropionate (DMSP) metabolism and dimethyl sulfoxide (DMSO) reduction were absent from “*Ca.* Endozoicomonas penghunesis,” but *E. acroporae* had a complete operon for DMSP metabolism, as reported in our previous study (36), as well as genes for DMSO reduction. The lack of a potent oxidative stress response gene repertoire could be the reason for the loss of abundance of “*Ca.* Endozoicomonas penghunesis” in the control (IC) and transplant samples (I→O) in summer (June-August). Furthermore, the robustness of *E. acroporae* in the control (OC) and transplant samples (O→I) throughout the year could be due to the species’ ability to remove oxidative stress (which increased in summer) more efficiently via DMSP metabolism and the presence of catalase activity. This was further confirmed by using the 2,2 diphenyl-1-picrylhydrazyl (DPPH) assay as a proxy for measuring the ROS scavenging ability, where *E. acroporae* was identified as a strong free radical scavenger compared to “*Ca.* Endozoicomonas penghunesis” in ambient (25°C) as well as higher temperature conditions (30°C) (Fig. S6).

10.1128/msystems.00359-22.6 FIG S6 Determination of DPPH radical scavenging activity of two *Endozoicomonas* species at 25°C and 30°C. The assay was performed after 24 h of incubation. All experiments were performed in triplicate, and data are expressed as means ± standard deviations. Differences between the mean from each two groups were calculated by two-tailed paired Student *t* test *P* value indicate statistical significance (*, *P* < 0.005; **, *P* < 0.0005). Download FIG S6, PDF file, 0.1 MB. Copyright © 2022 Tandon et al. 2022 Tandon et al. https://creativecommons.org/licenses/by/4.0/ This content is distributed under the terms of the Creative Commons Attribution 4.0 International license.

### Coral mortality in the Inner Bay.

We observed coral mortality exclusively in the Inner Bay for both IC and O→I samples (Fig. 2A and B, marked X). Grazing by *Drupella cornus* was also observed in the samples from the Inner Bay during the sampling in May and continued until December (Fig. S7).

10.1128/msystems.00359-22.7 FIG S7 *Drupella cornus*-nibbled Inner Bay *Acropora muricata* corals. (A) Coral *Acropora* sp. strain Colony attacked by *Drupella cornus* (red arrows). (B) Zoomed-in photo of panel A. (C) Another colony being attacked. (D) Zoom-in photo of panel C. Coral colonies in the Inner Bay only suffered from *Drupella cornus*, which nibbled coral, potentially causing bleaching and death. Download FIG S7, PDF file, 0.9 MB. Copyright © 2022 Tandon et al. 2022 Tandon et al. https://creativecommons.org/licenses/by/4.0/ This content is distributed under the terms of the Creative Commons Attribution 4.0 International license.

## DISCUSSION

This study aimed to test the differential capability of *Endozoicomonas*, one of the most dominant bacterial groups in the coral microbiome toward environmental change. We analyzed the microbiome dynamics of the common Indo-Pacific coral *A. muricata* over 9 months following a reciprocal transplant experiment at the finest resolution of zOTUs. We identified that different *Endozoicomonas* phylotypes in the *A. muricata* coral colonies belong to two dominant species, including a novel species, and have differential adaptation capabilities, with one species more resilient to environmental change than the other. Our results shed light on an often-neglected factor when determining variations in community composition: bacterial species/strains adapt differently when coral hosts are subjected to biotic and abiotic stressors. Furthermore, we also isolated, cultured, and sequenced a single chromosome-level genome of one of the dominant phylotypes belonging to a novel *Endozoicomonas* species, “*Ca.* Endozoicomonas penghunesis” 4G, to ascertain the ecological and functional role of this bacterium and add to the growing knowledge and genome data sets of this key microbe in coral reefs.

*Acropora muricata* microbiome was dominated by members of class *Gammaproteobacteria* (phylum *Proteobacteria*) (Silva v132 grouped *Gammaproteobacteria* into *Betaproteobacteria*), particularly by *Endozoicomonas-*related phylotype*s* (Fig. 2A and B and Fig. S2). Members of the genus *Endozoicomonas* are often found to be the dominant group in the microbiome of several coral species, e.g., members of *Acropora*, *Pocillopora*, and *Stylophora* (3, 25, 31, 42), and have been proposed to play a significant role in coral health and protection (2, 43) and coral sulfur cycling (36, 44). Another dominant bacterial group, *Simkaniaceae* (phylum *Chlamydiae*, class *Chlamydiae*), was described as an obligate intracellular bacterium, but its function has remained enigmatic (42, 45). Like *Endozoicomonas*, *Simkaniaceae*-related phylotypes were also recently found to be abundant in healthy corals from the reefs in Florida, but their abundance decreased in corals suffering from stony coral tissue loss disease (46). Members of class *Mollicutes*, particularly zOTUs related to *Entomoplasmatales* and *Mycoplasmatales*, are suggested to be mutualistic or commensal bacteria in temperate and deep-sea gorgonians and cold-water Scleractinia corals, but their specific function remains unknown (47, 48). Overall, the microbial community composition in the coral colonies of the control group remained stable throughout the experiment timeline, with only transient differences observed between them across sampling times (Fig. 2A and B).

Spatial and temporal fluctuations in the microbiome were observed throughout the experiment, and community structure (Fig. 2A) and ordination analysis showed that community varies across temporal and spatial scales (Fig. 3A). Various degrees of overlap between the samples from the same location suggested that the microbes show different scales of variability and that microbial structure is a function of the local environment. Previous studies have also shown that the microbiome varies spatially due to differences in the sites’ local environments (19, 49). Ocean currents are believed to have a homogenizing effect on the microbial communities, and the same coral species separated by hundreds to thousands of kilometers have been found to have similar microbiome compositions (1, 50). Our results were in contrast to this observation, as a high site-to-site variation was observed at a relatively small scale. One potential reason for this variation is high abiotic and anthropogenic pollution in the Inner Bay compared to the Outer Bay; similar results were obtained in earlier studies from the Red Sea (28) and Singapore (51).

Utilizing the zOTU approach for metabarcoding data analysis and focusing on the dominant bacterial genus, we identified that colonies of *A. muricata* from the Inner and Outer Bays were not dominated by a single *Endozoicomonas* phylotype but had several differentially abundant phylotypes associated with them (Fig. 2B). These results are similar to the multiple phylotypes that were reported to be dominant in colonies of the same coral genus as identified earlier (42). However, the dominant *Endozoicomonas* phylotypes identified in our study were different in coral colonies in the two locations, with Inner Bay colonies harboring a novel species “*Ca.* Endozoicomonas penghunesis” and Outer Bay colonies harboring *E. acroporae-*related phylotypes (Fig. 4A). This result was intriguing, as corals of the *Acropora* genus are known to have a strong influence on their microbial community composition (19). Another observation that arises from these results is how the single-nucleotide variation approach utilized to obtain ASVs or zOTUs potentially can lead to increased diversity (richness) estimates, which rely on ASV or zOTU counts, especially with bacterial groups known to have more than one copy of nonidentical 16S rRNA genes in their genome. In our study this is true in the case of the genus *Endozoicomonas*, as members of this genus are known to harbor more than one copy of 16S rRNA and complete genomes of Endozoicomonas montiporae CL-33^T^ have seven copies (52), similar to “*Ca.* Endozoicomonas penghunesis,” which are not all identical (Fig. S3); hence, we used a phylogenetic approach to assign taxonomy to these *Endozoicomonas* phylotypes.

Members of the coral holobiont potentially engage in complex interactions to maintain the health and fitness of the coral host, and external stressors could disturb these interactions by influencing the composition of the holobiont. To overcome the influence of the external stressors, a genomic adaptation of the holobiont members (in our case, bacteria) could play an important role in host survival, since a bacterium is likely to be not as well adapted to a new niche as resident strains, unless it has the genetic capability to mitigate the new stressors (53, 54). During our reciprocal transplant experiment, we observed that *E. acroporae*-related zOTUs remained dominant in the control (OC) and the transplanted samples (O→I), with only a change in the dominant zOTU from zOTU1 to zOTU2, whereas those related to “*Ca.* Endozoicomonas penghunesis” 4G were only dominant in April and May months in the control samples of the Inner Bay (IC) (Fig. 2B). There are a few possible reasons for this observation. One is that *E. acroporae* may be more resilient and better adapted to diverse conditions like increased oxidative stress during summers, as evident from the DPPH assay compared to “*Ca.* Endozoicomonas penghunesis” 4G. However, further investigation is required to say this with greater confidence, as the DPPH assay only measures polyphenolic compounds and, hence, cannot be taken as representative of the overall bacterial antioxidant capacity. Another potential explanation is the restructuring of the microbiome by the host *A. muricata*. The latter seems more plausible with evidence from recent studies on *Acropora*.

In addition, variation in the abundance of the different *Endozoicomonas* phylotypes is potentially analogous to the abundance of genotypes of different endosymbiotic algae *Symbiodinium*, often found in coral colonies (55). Several coral species can perform “symbiont shuffling or switching” to select for the more thermotolerant genotype of endosymbiotic algae in response to thermal stress (56–58). We observed microbial shuffling for *Endozoicomonas* phylotypes to a certain degree in our transplant samples. zOTU1 (potentially *E. acroporae* (Fig. 4A)) became the dominant *Endozoicomonas* phylotype in I→O samples, although zOTU7 and zOTU9 (potentially “*Ca.* Endozoicomonas penghunesis” 4G [Fig. 4A]) were the dominant phylotypes in IC samples (Fig. 2B). It is important to note here that no microbial shuffling was observed in the case of O→I versus OC samples, as phylogenetic analysis identified both zOTU1 and -2 as *E. acroporae* (Fig. 4A). Furthermore, the DPPH assay identified a higher potential for *E. acroporae* to scavenge ROS than “*Ca.* Endozoicomonas penghunesis” (Fig. S6), so shuffling microbial community might confer selective advantages to the coral host but further exploration is required.

Understanding the functional and ecological roles of coral-associated microbes in reefs has become critical to developing an intervention for coral reef protection, such as developing a coral probiotic (2, 59). These interventions require in-depth information about members of the coral holobiont. In the current study, we isolated, cultured, and sequenced the complete genome of a dominant *Endozoicomonas* phylotype identified in the metabarcoding data analysis. Phylogenetic analysis identified that the dominant zOTUs (zOTU1, zOTU2, zOTU10, zOTU11, zOTU13, and zOTU18) from the Outer Bay were closer to a previously characterized species, *E. acroporae* (60), whose genome was sequenced earlier (36, 61). On the other hand, the Inner Bay-dominant zOTUs (zOTU7, zOTU9, zOTU16, and zOTU17) were closest to the novel species “*Ca.* Endozoicomonas penghunesis” 4G isolated and characterized in this study. Genomic analysis of “*Ca.* Endozoicomonas penghunesis” revealed features similar to those of other *Endozoicomonas* species, i.e., the large genome size (~6.00 Mb), many coding genes (4,913), and complete pathways for essential amino acids, suggesting a free-living life stage. Furthermore, a comparative genomics study identified that *Endozoicomonas* species are capable of differential functional specificity, and different genotypes may play disparate metabolic roles in their hosts (37). This is true for sulfur metabolism, where *E. acroporae* is the only known *Endozoicomonas* species capable of metabolizing dimethylsulfoniopropionate (DMSP) to dimethylsulfide (DMS) (36). Genes for DMSP metabolism operon were not present in the genome of “*Ca.* Endozoicomonas penghunesis.” However, transporters (three copies) for glycine-betaine, another osmolyte, were identified in the genome of “*Ca.* Endozoicomonas penghunesis.” Other *Endozoicomonas* species have also been identified to have the ability to scavenge glycine-betaine through transporters (62), potentially alleviating oxidative stress. Identification of putative siroheme-dependent anaerobic sulfite reduction operon was interesting as this process facilitates growth under anaerobic conditions (B_12_-dependent anaerobic growth) by oxidizing 1,2-propanediol with tetrathionate as an electron acceptor (63). Physiological tests also showed that “*Ca.* Endozoicomonas penghunesis” is a facultative anaerobe; however, more functional evidence is required to confirm this outcome and the advantage (if any) it provides to the bacterium and coral host that maintains it.

The antioxidant-producing ability of marine invertebrate-associated bacteria had been wildly reported (64, 65). Living in highly variable environments, marine bacteria have developed different strategies to protect themselves from reduced oxygen intermediates and increase environmental adaptability (66). Compared to “*Ca.* Endozoicomonas penghunesis” 4G, *E. acroporae* Acr-14^T^ showed stronger free radical scavenging ability at higher temperatures, no matter in cell lysate or supernatant (see Fig. S6 in the supplemental material). Based on our results, we suggest that the dominance of *E. acroporae-*related phylotypes after reciprocal is due to higher antioxidant abilities, especially in summer. However, the interactions between coral and bacteria are complicated, and it is hard to clarify the specific function of bacterial antioxidants and their relationship with niche selection. Therefore, more focused studies are required to discern the putative benefits of the coral microbiome concerning oxidative stress management for the host.

We observed coral mortality during our experiment exclusively in the Inner Bay; the corallivorous snail *Drupella cornus*, which exclusively feeds on living tissue, grazed there (Fig. S7). These gastropods occur throughout the shallow waters of the Indo-Pacific region (67). Outbreaks of this corallivorous marine gastropod have been recorded in different parts of the Gulf of Eilat, Israel (68), and the Great Barrier Reef, Australia (69). Coral feeding gastropods of *Drupella* species show a strong preference for preying on *Acroporids* (70) and are known to be efficient vectors for brown band disease in corals (69, 71). Although no *Drupella* species outbreaks to date have been recorded in Taiwan’s coral reefs and no visible signs of brown band disease were observed in our study, it is important to keep monitoring the corals in the Penghu Archipelago for signs of climate change and disease outbreaks in the near future.

### Conclusions.

A variety of factors, many of which are external, are known to influence the coral microbiome composition and its dynamics. However, an important internal factor, the adaptation capability of microbiome members, which governs the survival of a bacterium in a niche, has been overlooked. Using a combination of metabarcoding, genomic, and comparative genomic approaches, we showed that members of the dominant bacterial group *Endozoicomonas* are capable of sustaining and proliferating in a new niche following a reciprocal transplant experiment. Our ability to isolate and culture one of the dominant bacterial species, “*Ca.* Endozoicomonas penghunesis” 4G, builds on our knowledge of these important bacterial groups in the coral holobiont. Furthermore, we address critical aspects of using zOTUs/ASVs to estimate bacterial richness using metabarcoding data, which can result in often falsely inflated diversity estimates, especially in the case of microbes harboring more than one copy of nonidentical 16S rRNA gene, e.g., *Endozoicomonas*. In summary, we conclude that different members of the coral holobiont belonging to the same bacterial group can have differential adaptation capabilities, and this internal factor should also be considered when devising interventions to protect coral reefs, like developing a coral probiotic.

## MATERIALS AND METHODS

### Study design and experimental setup.

Five colonies of *Acropora muricata* (40 by 40 cm) were collected at a depth of 3 m from the Outer Bay (O) (N23° 33.097' E119° 38.335′) and Inner Bay (I) (N23° 31.853′ E119° 33.629') along the reef adjacent to the coast of the Penghu Archipelago, Taiwan (Fig. 1A). Mother coral colonies were first collected in April. These acted as controls for the native coral microbiome in the study sites (Fig. 1A). Later, mother colonies were fragmented into two halves (approximately 20 by 20 cm each). Coral fragments from mother colonies were either cross swapped (I→O or O→I) or transplanted in their original location (IC or OC) (Fig. 1B). Coral fragments from each colony that remained in their original location acted as controls to measure any change in the microbiome due to the transplant procedure and change in the microbiome based on colony age and experimental time. Coral fragments were glued onto the reef with epoxy putty.

### Sampling timeline and sample collection.

Study sites were visited every month from April to August 2018 and then in December 2018 to check the status of transplanted fragments and collect samples. In total, we collected 122 samples, including seawater samples (1 liter) at each time point and location. Two- by 2-cm fragments were taken from each colony, rinsed with filtered seawater, and stored in 99% ethanol at −20°C before DNA extraction.

### DNA extraction and 16S rRNA gene amplicon sequencing.

Frozen coral fragments were sprayed (70 lb/in^2^) with ~15 ml 1× TE buffer (10 mM Tris-HCl, 1 mM EDTA, pH 8) and then placed into sterile Ziploc bags. Total DNA was extracted using a modified cetyltrimethylammonium bromide (CTAB) method (72). Coral tissue sample DNA was extracted with conventional chloroform-isoamyl alcohol (24:1) and phenol-chloroform–isoamyl alcohol (25:24:1) step and isopropanol precipitation method. The DNA pellet was rinsed with 70% ethanol and then dissolved in 50 μl double-distilled water and stored at −20°C. DNA concentration was determined using a NanoDrop 1000 spectrophotometer (Thermo Fisher Scientific, Waltham, MA) and Quant-iT dsDNA HS (high-sensitivity) assay kit. Seawater samples were processed similarly with the modified CTAB method (72).

For DNA library construction, 968F (5′-AAC GCG AAG AAC CTT AC-3′) (73) and 1391R (5′-ACG GGC GGT GWG TRC-3′) (74) universal primers were used to amplify the bacterial V6-V8 hypervariable region of the 16S rRNA gene from the total DNA from samples using PCR. For PCRs, 50 to 150 ng of template DNA was used. PCR was performed in 50-μl reaction volumes, consisting of 1.5 U TaKaRa *Ex Taq* (TaKaRa Bio, Otsu, Japan), 1× TaKaRa *Ex Taq* buffer, 0.2 mM deoxynucleotide triphosphate mixture (dNTPs), 0.2 mM forward and reverse primers, and template DNA. The PCR conditions consisted of an initial denaturing step at 95°C for 5 min, followed by 30 cycles at 94°C for 30 s, 52°C for 20 s, and 72°C for 45 s and a final extension at 72 °C for 10 min. The amplified product was visually confirmed using 1.5% agarose gel with a 5-μl PCR product. Target bands (~420 bp) were cut and eluted using a QIAEX II gel extraction kit (Qiagen, Valencia, CA, USA).

Each bacterial V6-V8 amplicon was tagged with a unique barcode sequence by designing tag primers with 4-base overhangs at 5′ ends. The sample-specific tagging reaction was performed with a 5-cycle PCR, with a reaction program of initial denaturation at 94°C for 3 min, followed by denaturation at 94°C for 30 s, annealing at 52°C for 20 s, extension at 72°C for 45 s, and a final extension at 72°C for 10 min. The amplified product was purified using the QIAquick PCR purification kit (Qiagen, Valencia, CA). Purified products were pooled into four independent libraries and sequenced with Illumina MiSeq paired-end sequencing (2 × 300 bp) at Yourgene Biosciences, Taiwan. No kit and PCR negative controls were sequenced in this study.

### Sequence data processing and analysis.

Paired-end raw reads obtained from Illumina sequencing were merged using USEARCH v11 (75) with the parameters minovlen = 16, maxdiffs = 30, and pctid = 80. Merged reads were sorted, quality filtered, and trimmed using Mothur v1.3.81 (76). Reads 400 to 470 bp long with an average quality of >25 were kept. Chimeric reads were inspected and eliminated with UCHIME (77) by USEARCH v11. Qualified reads were retained for subsequent analysis. High-quality reads were denoised using UNOISE3 (78), and zero-radius operational taxonomic units (zOTUs), which are equivalent to exact sequence variants, were obtained. The denoised sequences were aligned against the SILVA128 (79, 80) rRNA database for a taxonomic assignment up to the genus level using Mothur on a per-sample basis with a pseudobootstrap cutoff of 80%.

### Statistical analyses.

All statistical analyses and graphs were generated in R (R Core Team, 2020). Stacked bar plots were obtained by converting absolute abundance profiles into relative abundances. Abundance profiles were processed with the R packages phyloseq (81), vegan (82), ggplot2 (83), pheatmap (84), and microbiomeMarker (85) for downstream analyses and visualization. Alpha diversity analysis was conducted after rarifying the samples to an even depth of 5,704 reads using the estimate_richness function from phyloseq. Alpha diversity metrics were compared using analysis of variance (ANOVA) and Tukey’s *post hoc* tests using vegan package *P* value correction for multiple testing. Multivariate analysis was performed after square root transforming the zOTU count data. Betadisper function was used to calculate the multivariate dispersion of samples (Bray-Curtis distance) between sample groups. Homogeneity of multivariate dispersion was tested with ANOVA. Nonmultidimensional scaling (nMDS) was performed to compare community compositions using the Bray-Curtis distance metric between sample groups. Permutational multivariate analysis of variance (PERMANOVA) with the “adonis” function (with 9,999 permutations) was used to statistically test for differences in community compositions between the back and cross transplant samples for each location as dispersion was significantly different between groups. Linear discriminant analysis effect size (LEfSe) implemented in the microbiomeMarker package in R was used to identify shifts in zOTUs between back- and cross-transplant samples for each location with a log Linear discriminant analysis (LDA) cutoff of 3 (Kruskal-Wallis test, *P* < 0.05). *z* score-transformed abundance profiles of marker zOTUs identified from LEfSe were visualized with a heatmap via pheatmap.

### Environmental parameters.

The water temperatures of the Outer (O) and Inner (I) Bays of the Penghu Archipelago, Taiwan, were obtained from May 2018 through December 2018 using temperature data loggers (HOBO Pendant, Onset Corp.) located ~3 m deep, close to target colonies, and recording temperatures every 30 min. Abiotic factors, including NH_3_, NO_3_, and PO_4_, were measured with a LaMotte 1910 SMART 3 colorimeter; pH was measured with a HORIBA LAQUA act water quality meter, and salinity was measured with an ATAGO master refractometer.

### Bacteria isolation and culturing.

“*Ca.* Endozoicomonas penghunesis” 4G was isolated from the coral *Acropora muricata* off the coast of the Inner Bay, Penghu Archipelago, Taiwan (GPS location, N23° 31.851′ E119° 33.631′). Coral tissue and mucus were sprayed with TE buffer (10 mM Tris-HCl, 1 mM EDTA, pH 8) and serially diluted to 10^−4^. All dilutions were plated on modified marine broth version 4 (MMBv4 agar) (52) and incubated at 25°C. Each colony was screened first by the following primers: bacterial universal forward 27F (5'-AGA GTT TGA TCM TGG CTC AG-3′) and *Endozoicomonas*-specific reverse En771R (5′-TCA GTG TCA RRC CTG AGT GT-3′) (86). *Endozoicomonas* species 16S rRNA gene V1-V4 region was PCR amplified by 35 cycles with a denaturing step at 94 °C for the 30 s, followed by annealing at 54 °C for 30 s and an extension step at 72 °C for 45 s. PCR product was checked on a 1.5% agarose gel after electrophoresis. All samples with bands ~750 bp long were then subcultured in MMB medium. Full-length 16S rRNA genes were amplified by universal bacterial primer 27F (5′-AGA GTT TGA TCC TGG CTC AG-3′) and 1541R (5′-AAG GAG GTG ATC CAG CC-3′). The full-length 16S rRNA PCR was amplified using 30 cycles with a denaturing step at 95°C for 30 s, annealing at 55°C for 30 s, and a final extension at 72°C for 90 s. Amplified products with target bands (~1,465 bp) were cut and later sequenced by Sanger sequencing (3730 DNA analyzer; Thermo) from Genomics, Taipei, Taiwan. Chromatograms obtained were manually checked and sequences were trimmed. The final length of the high-quality trimmed sequence was ~600 bp. Sequences with ≤98% identity to 16S rRNA genes of type strains from genus *Endozoicomonas* were deemed new candidates for novel *Endozoicomonas* species.

### Physiological characterization.

“*Ca.* Endozoicomonas penghunesis” 4G was cultivated on MMBv4 medium (52) (see Table S1 in the supplemental material) for the enrichment, and a broad range of physiological characterizations was performed. The optimum salinity was tested on MMB medium with NaCl concentrations adjusted as required (0.5% and 1.0 to ~4.0%, wt/vol, in increments of 1.0%). The growth temperature range was tested at 4°C and 10 to 40°C (at 5°C intervals). The pH tolerance was determined using the following buffers: pH 4.0 to 7.0, HCl; pH 7.0 to 10.0, NaOH (at 1.0 pH unit intervals).

10.1128/msystems.00359-22.8 TABLE S1 Recipe of modified marine broth (MMB). Download Table S1, DOCX file, 0.02 MB. Copyright © 2022 Tandon et al. 2022 Tandon et al. https://creativecommons.org/licenses/by/4.0/ This content is distributed under the terms of the Creative Commons Attribution 4.0 International license.

Three physiological tests (pH, salinity, and temperature) were measured based on the turbidity (at the optical density at 600 nm) of cultures grown at different pH values, NaCl concentrations, and temperatures, respectively. Commercial API 20NE kits (bioMérieux, France) were used to test the ability to metabolize different carbon substrates per the manufacturer’s protocol. Additional carbon utilization was evaluated in the modified marine medium (see details in Table S2). Bacterial motility was tested in marine broth semisolid agar (0.5% agarose). The Gram stain kit (Fluka, England) was used to distinguish bacterial Gram reactions. Relation to oxygen was determined after incubating “*Ca.* Endozoicomonas penghunesis” 4G on MMB agar in the 2.5-liter Oxoid AnaroGen system (Thermo) and cultured at 25°C for 7 days. Oxidase and catalase activity was tested independently by adding 35% H_2_O_2_ and 0.1% tetramethyl–phenylenediamine dihydrochloride, respectively. An antibiotic sensitivity test was performed after spreading bacteria on an MMB plate, with each disc containing different antibiotics (10 μg streptomycin and 10 μg ampicillin). The results were observed after 5 days of incubation at 25°C, and sensitivity was measured based on the distance from the discs to the edge of the clear zone. Bacteria were scored as sensitive if the diameter was greater than 2 mm, slightly sensitive if the diameter was 1 to 2 mm, and resistant otherwise.

### Morphological characterization.

The morphology of “*Ca.* Endozoicomonas penghunesis” 4G, including colony shape and color, was observed by a stereomicroscope (EZ4; Leica, Germany). General cell structure and cell inner structure were studied by transmission electron microscopy (TEM). The bacterial shape on a single colony was observed by scanning electron microscopy (SEM). TEM and SEM observations were made after bacteria were cultured in MMB for 1 day and MMB agar (1.5%) for 3 days, respectively. Colonies were incubated at 25 °C.

For the “*Ca.* Endozoicomonas penghunesis” 4G thin section, bacteria were first centrifuged at 2,500 × *g* for 5 min, and bacterial pellets were collected and fixed in 2.5% glutaraldehyde and 4% paraformaldehyde in a 0.1 M sodium phosphate buffer (pH 7.0) at room temperature for 1 h. After three 20-min buffer rinses, the samples were postfixed in 1% OsO_4_ in the same buffer for 1 h at room temperature and then rinsed again as described above. Samples were dehydrated in an alcohol series, embedded in Spurr’s resin (EMS), and sectioned with a Leica EM UC6 ultramicrotome (Leica, Germany). The ultrathin sections (70 to 90 nm) were stained with 5% uranyl acetate in 50% methanol and 0.4% lead citrate in 0.1 N sodium hydroxide. An FEI G2 Tecnai Spirit Twin TEM was used at 80 kV for viewing, and images were captured with a Gatan Orius charge-coupled device (CCD) camera.

The colony of “*Ca.* Endozoicomonas penghunesis” 4G was observed using cryo-SEM (FEI Quanta 200 SEM/Quorum Cryo System PP2000TR). The MMBv4 agar plate containing a single colony of “*Ca.* Endozoicomonas penghunesis” 4G was sectioned into 1 mm by 1 mm, loaded onto the medium-containing stub, and then frozen with liquid nitrogen slush. The frozen sample was transferred to the sample preparation chamber at −160°C. After 5 min, the temperature was raised to −85°C, and the samples were etched for 20 min. After coating at −130°C, the samples were transferred to the SEM chamber and observed at −160°C and 20 kV.

The general cell morphology was studied by negative staining and observed under TEM. Bacteria were enriched in MMB for 1 day before adding a fixative solution (2.5% glutaraldehyde plus 4% paraformaldehyde–0.1 M PBS) at 37°C for 10 min. To reduce the background signal of TEM observation, MMB was replaced first by PBS and then by sterilized H_2_O twice, and the bacteria were mounted onto grow-discharge carbon-Formvar grids. Bacteria were stained by 2% phosphotungstate for 1 s, and finally the sample was rinsed with sterilized H_2_O twice and viewed under an FEI G2 Tecnai Spirit Twin TEM at 80 KV. The images were then captured with a Gatan Orius CCD camera.

### DPPH radical scavenging assay.

The ability of free radical scavenging of two bacterial species was tested by a 2,2 diphenyl-1-picrylhydrazyl (DPPH) assay. The two *Endozoicomonas* species were incubated in MMbv4 medium with 0.1% glucose at 25°C and 30°C for 24 h, and the bacterial supernatant was harvested by centrifugation at 13,000 rpm for 5 min at 4°C. To collect cell lysate, cell pellets were resuspended by 0.8 ml 50 mM Tris-HCl lysis buffer (50 mM HEPES, 2% Triton X-100, 5% glycerol, and 40 mg 180-μm plastic beads) and loaded into a 2-mL O-ring-capped plastic microcentrifuge tube. The cell pellets with lysis buffer were vortexed at maximum intensity for 5 min and incubated on ice for 1 min. The step was repeated twice. After that, the cell lysates were centrifuged at 13,000 rpm for 10 min at 4°C to pellet down the cell debris and beads. The hydrogen atom donating ability of bacterial cultural supernatant and bacterial cell lysates was determined by decolorization of methanol solution of DPPH from violet color to yellow color in the presence of antioxidants. The 0.2 mM DPPH solution in 100% methanol was prepared, and 100 μl of the sample (supernatant or cell lysates) was mixed with 100 μl DPPH in 96-well plates in triplicate. Blanks were 100 μl sample mixed with 100 μl DPPH solvent (100% methanol), and control was 100 μl sample solvent (MMbv4 as the supernatant solvent and Tris-HCl lysis buffer as cell lysate sample) mixed with 100 μl DPPH solution. The reaction mixtures were incubated in the dark at 25°C for 30 min. The absorbance of the mixture was measured by a microplate reader (SpectraMax M2; Molecular Devices) at 517 nm. The DPPH scavenging activity was defined as

(1) $$\text{DPPH free radical scavenging }\left(\text{\%}\right)=1-\frac{{\text{ABS}}_{\text{sample}}{-\text{ABS}}_{\text{blank}}}{{\text{ABS}}_{\text{control}}{-\text{ABS}}_{\text{blank}}}\times 100$$

where ABS_sample_ is the absorbance of the mixture of DPPH solution with samples, ABS_blank_ is the absorbance of the mixture of samples and DPPH solvent, and ABS_control_ is the absorbance of the mixture of sample solvent and DPPH solution.

### Phylogenetic analysis of *Endozoicomonas* species zOTUs.

To phylogenetically place the dominant *Endozoicomonas* zOTUs identified and the 16S rRNA gene of “*Ca.* Endozoicomonas penghunesis” 4G and to identify their closest neighbor within genus *Endozoicomonas* and its cultured isolates, representative 16S rRNA sequences from type strains (12 total) and one outgroup Halospina denitrificans HGD were downloaded from the NCBI taxonomy database (https://www.ncbi.nlm.nih.gov/taxonomy). Sequences were aligned using the RNA homology search tool cmalign (87) from the infernal package, and the CM models for domain bacteria were acquired from the rfam database (88). A maximum-likelihood phylogeny tree was built using the IQ-TREE web server (89) with 1,000 bootstraps and the best model selection enabled (best model, K2P+I+G4). The tree was finally visualized and edited in the laboratory-licensed version of iTOL v4 (90).

### Long- and short-read paired-end sequencing and genome assembly.

Long reads obtained from nanopore sequencing were first quality checked with nanoqc (91) and only high-quality paired-end reads were used for genome assembly using metaFlye (92) with default settings. Illumina reads (2× 300) were first quality checked with FastQC (https://www.bioinformatics.babraham.ac.uk/projects/fastqc/), and then the adapters were removed and the reads trimmed with AdapterRemoval v2 (93). High quality (phred, >30) and trimmed paired-end reads were used to polish the crude nanopore assembly with four rounds of pilon (94) with default settings.

### Genome annotation.

The assembled genome was first checked for completeness, contamination, and heterogeneity using CheckM (40). The *E. acroporae* Acr-14^T^ genome (61) was assembled previously in our laboratory. Protein predictions in two *Endozoicomonas* species were performed with Prodigal in Prokka (95) with default settings to keep gene calls preserved for further functional categories analysis. A rapid annotation using subsystem technology (RAST) server (96) was used to obtain higher-order subsystem-level features. The “reconstruct pathway” approach in blastKOALA v2.2 (97) was used to obtain KEGG Ontology (KO) terms and in-depth annotation of the proteome. CRISPRcasFinder (98) was used to access the CRISPR-spacer. Eukaryote-like proteins (ELPs) were searched from a Batch Web-CD search against the CDD database (99), with minimum E value of 1e−5 and maximum hit number set to 50. Circular genomic map of “*Ca.* Endozoicomonas penghunesis” 4G was visualized by CGView Server beta (100). Average nucleotide identity (ANI) analysis was performed to delineate the genome identity of “*Ca.* Endozoicomonas penghunesis” 4G against other *Endozoicomonas* species genomes which are publicly available from National Center for Biotechnology Information (NCBI) using the ANI calculator from the enveomics collection (http://enve-omics.ce.gatech.edu/ani/) (101).

### Data availability.

All sequencing data generated in the manuscript was submitted under the BioProject no. PRJNA758232, and the “*Ca.* Endozoicomonas penghunesis” genome was made available under GenBank accession no. SAMN21016876.

## References

[1] Rohwer F, Seguritan V, Azam F, Knowlton N. 2002. Diversity and distribution of coral-associated bacteria. Mar Ecol Prog Ser 243:1–10. doi:10.3354/meps243001.

[2] Peixoto RS, Rosado PM, Leite DCA, Rosado AS, Bourne DG. 2017. Beneficial microorganisms for corals (BMC): proposed mechanisms for coral health and resilience. Front Microbiol 8:341. doi:10.3389/fmicb.2017.00341. 28326066 PMC5339234

[3] van Oppen MJH, Blackall LL. 2019. Coral microbiome dynamics, functions and design in a changing world. Nat Rev Microbiol 17:557–567. doi:10.1038/s41579-019-0223-4. 31263246

[4] Blackall LL, Wilson B, van Oppen MJH. 2015. Coral-the world’s most diverse symbiotic ecosystem. Mol Ecol 24:5330–5347. doi:10.1111/mec.13400. 26414414

[5] Bourne DG, Munn CB. 2005. Diversity of bacteria associated with the coral Pocillopora damicornis from the Great Barrier Reef. Environ Microbiol 7:1162–1174. doi:10.1111/j.1462-2920.2005.00793.x. 16011753

[6] Sweet MJ, Croquer A, Bythell JC. 2011. Bacterial assemblages differ between compartments within the coral holobiont. Coral Reefs 30:39–52. doi:10.1007/s00338-010-0695-1.

[7] Li J, Chen Q, Long L-J, Dong J-D, Yang J, Zhang S. 2014. Bacterial dynamics within the mucus, tissue and skeleton of the coral Porites lutea during different seasons. Sci Rep 4:7320. doi:10.1038/srep07320. 25475855 PMC4256709

[8] Glasl B, Herndl GJ, Frade PR. 2016. The microbiome of coral surface mucus has a key role in mediating holobiont health and survival upon disturbance. ISME J 10:2280–2292. doi:10.1038/ismej.2016.9. 26953605 PMC4989324

[9] Marchioro GM, Glasl B, Engelen AH, Serrão EA, Bourne DG, Webster NS, Frade PR. 2020. Microbiome dynamics in the tissue and mucus of acroporid corals differ in relation to host and environmental parameters. PeerJ 8:e9644. doi:10.7717/peerj.9644. 32874778 PMC7439960

[10] Yang S-H, Tseng C-H, Huang C-R, Chen C-P, Tandon K, Lee STM, Chiang P-W, Shiu J-H, Chen CA, Tang S-L. 2017. Long-term survey is necessary to reveal various shifts of microbial composition in corals. Front Microbiol 8:1094. doi:10.3389/fmicb.2017.01094. 28659905 PMC5468432

[11] Pollock FJ, McMinds R, Smith S, Bourne DG, Willis BL, Medina M, Thurber RV, Zaneveld JR. 2018. Coral-associated bacteria demonstrate phylosymbiosis and cophylogeny. Nat Commun 9:4921. doi:10.1038/s41467-018-07275-x. 30467310 PMC6250698

[12] Agostini S, Suzuki Y, Higuchi T, Casareto BE, Yoshinaga K, Nakano Y, Fujimura H. 2012. Biological and chemical characteristics of the coral gastric cavity. Coral Reefs 31:147–156. doi:10.1007/s00338-011-0831-6.

[13] Marcelino VR, van Oppen MJ, Verbruggen H. 2018. Highly structured prokaryote communities exist within the skeleton of coral colonies. ISME J 12:300–303. doi:10.1038/ismej.2017.164. 29053151 PMC5739017

[14] Yang S-H, Tandon K, Lu C-Y, Wada N, Shih C-J, Hsiao SS-Y, Jane W-N, Lee T-C, Yang C-M, Liu C-T, Denis V, Wu Y-T, Wang L-T, Huang L, Lee D-C, Wu Y-W, Yamashiro H, Tang S-L. 2019. Metagenomic, phylogenetic, and functional characterization of predominant endolithic green sulfur bacteria in the coral Isopora palifera. Microbiome 7:3. doi:10.1186/s40168-018-0616-z. 30609942 PMC6320609

[15] Ricci F, Fordyce A, Leggat W, Blackall LL, Ainsworth T, Verbruggen H. 2021. Multiple techniques point to oxygenic phototrophs dominating the Isopora palifera skeletal microbiome. Coral Reefs 40:275–282. doi:10.1007/s00338-021-02068-z.

[16] Littman RA, Willis BL, Pfeffer C, Bourne DG. 2009. Diversities of coral-associated bacteria differ with location, but not species, for three acroporid corals on the Great Barrier Reef. FEMS Microbiol Ecol 68:152–163. doi:10.1111/j.1574-6941.2009.00666.x. 19302548

[17] Kvennefors ECE, Sampayo E, Ridgway T, Barnes AC, Hoegh-Guldberg O. 2010. Bacterial communities of two ubiquitous Great Barrier Reef corals reveals both site- and species-specificity of common bacterial associates. PLoS One 5:e10401. doi:10.1371/journal.pone.0010401. 20454460 PMC2861602

[18] Morrow KM, Moss AG, Chadwick NE, Liles MR. 2012. Bacterial associates of two Caribbean coral species reveal species-specific distribution and geographic variability. Appl Environ Microbiol 78:6438–6449. doi:10.1128/AEM.01162-12. 22773636 PMC3426691

[19] Dunphy CM, Gouhier TC, Chu ND, Vollmer SV. 2019. Structure and stability of the coral microbiome in space and time. Sci Rep 9:6785. doi:10.1038/s41598-019-43268-6. 31043671 PMC6494856

[20] Epstein HE, Smith HA, Cantin NE, Mocellin VJL, Torda G, van Oppen MJH. 2019. Temporal variation in the microbiome of Acropora coral species does not reflect seasonality. Front Microbiol 10:1775. doi:10.3389/fmicb.2019.01775. 31474944 PMC6706759

[21] Bourne D, Iida Y, Uthicke S, Smith-Keune C. 2008. Changes in coral-associated microbial communities during a bleaching event. ISME J 2:350–363. doi:10.1038/ismej.2007.112. 18059490

[22] Zaneveld JR, Burkepile DE, Shantz AA, Pritchard CE, McMinds R, Payet JP, Welsh R, Correa AMS, Lemoine NP, Rosales S, Fuchs C, Maynard JA, Thurber RV. 2016. Overfishing and nutrient pollution interact with temperature to disrupt coral reefs down to microbial scales. Nat Commun 7:11833. doi:10.1038/ncomms11833. 27270557 PMC4899628

[23] Ziegler M, Seneca FO, Yum LK, Palumbi SR, Voolstra CR. 2017. Bacterial community dynamics are linked to patterns of coral heat tolerance. Nat Commun 8:14213. doi:10.1038/ncomms14213. 28186132 PMC5309854

[24] Shiu J-H, Keshavmurthy S, Chiang P-W, Chen H-J, Lou S-P, Tseng C-H, Justin Hsieh H, Allen Chen C, Tang S-L. 2017. Dynamics of coral-associated bacterial communities acclimated to temperature stress based on recent thermal history. Sci Rep 7:14933. doi:10.1038/s41598-017-14927-3. 29097716 PMC5668310

[25] Maher RL, Schmeltzer ER, Meiling S, McMinds R, Ezzat L, Shantz AA, Adam TC, Schmitt RJ, Holbrook SJ, Burkepile DE, Vega Thurber R. 2020. Coral microbiomes demonstrate flexibility and resilience through a reduction in community diversity following a thermal stress event. Front Ecol Evol 8:356. doi:10.3389/fevo.2020.555698.

[26] Wang L, Shantz AA, Payet JP, Sharpton TJ, Foster A, Burkepile DE. 2018. Corals and their microbiomes are differentially affected by exposure to elevated nutrients and a natural thermal anomaly. Front Mar Sci doi:10.3389/fmars.2018.00101.

[27] Gignoux-Wolfsohn SA, Aronson FM, Vollmer SV. 2017. Complex interactions between potentially pathogenic, opportunistic, and resident bacteria emerge during infection on a reef-building coral. FEMS Microbiol Ecol 93:fix080. doi:10.1093/femsec/fix080. 28637338

[28] Ziegler M, Roik A, Porter A, Zubier K, Mudarris MS, Ormond R, Voolstra CR. 2016. Coral microbial community dynamics in response to anthropogenic impacts near a major city in the central Red Sea. Mar Pollut Bull 105:629–640. doi:10.1016/j.marpolbul.2015.12.045. 26763316

[29] Osman EO, Suggett DJ, Voolstra CR, Pettay DT, Clark DR, Pogoreutz C, Sampayo EM, Warner ME, Smith DJ. 2020. Coral microbiome composition along the northern Red Sea suggests high plasticity of bacterial and specificity of endosymbiotic dinoflagellate communities. Microbiome 8:8. doi:10.1186/s40168-019-0776-5. 32008576 PMC6996193

[30] La Rivière M, Garrabou J, Bally M. 2015. Evidence for host specificity among dominant bacterial symbionts in temperate gorgonian corals. Coral Reefs 34:1087–1098. doi:10.1007/s00338-015-1334-7.

[31] Ziegler M, Grupstra CGB, Barreto MM, Eaton M, BaOmar J, Zubier K, Al-Sofyani A, Turki AJ, Ormond R, Voolstra CR. 2019. Coral bacterial community structure responds to environmental change in a host-specific manner. Nat Commun 10:3092. doi:10.1038/s41467-019-10969-5. 31300639 PMC6626051

[32] Goldsmith DB, Kellogg CA, Morrison CL, Gray MA, Stone RP, Waller RG, Brooke SD, Ross SW. 2018. Comparison of microbiomes of cold-water corals Primnoa pacifica and Primnoa resedaeformis, with possible link between microbiome composition and host genotype. Sci Rep 8:12383. doi:10.1038/s41598-018-30901-z. 30120375 PMC6098105

[33] Glasl B, Smith CE, Bourne DG, Webster NS. 2019. Disentangling the effect of host-genotype and environment on the microbiome of the coral Acropora tenuis. PeerJ 7:e6377. doi:10.7717/peerj.6377. 30740275 PMC6368029

[34] Dubé CE, Ziegler M, Mercière A, Boissin E, Planes S, Bourmaud CA-F, Voolstra CR. 2021. Naturally occurring fire coral clones demonstrate a genetic and environmental basis of microbiome composition. Nat Commun 12:6402. doi:10.1038/s41467-021-26543-x. 34737272 PMC8568919

[35] Neave MJ, Rachmawati R, Xun L, Michell CT, Bourne DG, Apprill A, Voolstra CR. 2017. Differential specificity between closely related corals and abundant Endozoicomonas endosymbionts across global scales. ISME J 11:186–200. doi:10.1038/ismej.2016.95. 27392086 PMC5335547

[36] Tandon K, Lu C-Y, Chiang P-W, Wada N, Yang S-H, Chan Y-F, Chen P-Y, Chang H-Y, Chiou Y-J, Chou M-S, Chen W-M, Tang S-L. 2020. Comparative genomics: dominant coral-bacterium Endozoicomonas acroporae metabolizes dimethylsulfoniopropionate (DMSP). ISME J 14:1290–1303. doi:10.1038/s41396-020-0610-x. 32055028 PMC7174347

[37] Neave MJ, Michell CT, Apprill A, Voolstra CR. 2017. Endozoicomonas genomes reveal functional adaptation and plasticity in bacterial strains symbiotically associated with diverse marine hosts. Sci Rep 7:40579. doi:10.1038/srep40579. 28094347 PMC5240137

[38] Ribas-Deulofeu L, Denis V, De Palmas S, Kuo C-Y, Hsieh HJ, Chen CA. 2016. Structure of benthic communities along the Taiwan latitudinal gradient. PLoS One 11:e0160601. doi:10.1371/journal.pone.0160601. 27513665 PMC4981444

[39] Hsieh HJ, Hsien Y-L, Jeng M-S, Tsai W-S, Su W-C, Chen CA. 2008. Tropical fishes killed by the cold. Coral Reefs 27:599–599. doi:10.1007/s00338-008-0378-3.

[40] Parks DH, Imelfort M, Skennerton CT, Hugenholtz P, Tyson GW. 2015. CheckM: assessing the quality of microbial genomes recovered from isolates, single cells, and metagenomes. Genome Res 25:1043–1055. doi:10.1101/gr.186072.114. 25977477 PMC4484387

[41] Bowers RM, Kyrpides NC, Stepanauskas R, Harmon-Smith M, Doud D, Reddy TBK, Schulz F, Jarett J, Rivers AR, Eloe-Fadrosh EA, Tringe SG, Ivanova NN, Copeland A, Clum A, Becraft ED, Malmstrom RR, Birren B, Podar M, Bork P, Weinstock GM, Garrity GM, Dodsworth JA, Yooseph S, Sutton G, Glöckner FO, Gilbert JA, Nelson WC, Hallam SJ, Jungbluth SP, Ettema TJG, Tighe S, Konstantinidis KT, Liu W-T, Baker BJ, Rattei T, Eisen JA, Hedlund B, McMahon KD, Fierer N, Knight R, Finn R, Cochrane G, Karsch-Mizrachi I, Tyson GW, Rinke C, Lapidus A, Meyer F, Yilmaz P, Parks DH, Eren AM, Genome Standards Consortium, et al. 2017. Minimum information about a single amplified genome (MISAG) and a metagenome-assembled genome (MIMAG) of bacteria and archaea. Nat Biotechnol 35:725–731. doi:10.1038/nbt.3893. 28787424 PMC6436528

[42] Damjanovic K, Blackall LL, Peplow LM, van Oppen MJH. 2020. Assessment of bacterial community composition within and among Acropora loripes colonies in the wild and in captivity. Coral Reefs 39:1245–1255. doi:10.1007/s00338-020-01958-y.

[43] Neave MJ, Apprill A, Ferrier-Pagès C, Voolstra CR. 2016. Diversity and function of prevalent symbiotic marine bacteria in the genus Endozoicomonas. Appl Microbiol Biotechnol 100:8315–8324. doi:10.1007/s00253-016-7777-0. 27557714 PMC5018254

[44] Raina J-B, Tapiolas D, Willis BL, Bourne DG. 2009. Coral-associated bacteria and their role in the biogeochemical cycling of sulfur. Appl Environ Microbiol 75:3492–3501. doi:10.1128/AEM.02567-08. 19346350 PMC2687302

[45] Collingro A, Tischler P, Weinmaier T, Penz T, Heinz E, Brunham RC, Read TD, Bavoil PM, Sachse K, Kahane S, Friedman MG, Rattei T, Myers GSA, Horn M. 2011. Unity in variety–the pan-genome of the Chlamydiae. Mol Biol Evol 28:3253–3270. doi:10.1093/molbev/msr161. 21690563 PMC3247790

[46] Meyer JL, Castellanos-Gell J, Aeby GS, Häse CC, Ushijima B, Paul VJ. 2019. Microbial community shifts associated with the ongoing stony coral tissue loss disease outbreak on the Florida reef tract. Front Microbiol 10:2244. doi:10.3389/fmicb.2019.02244. 31608047 PMC6769089

[47] Gray MA, Stone RP, McLaughlin MR, Kellogg CA. 2011. Microbial consortia of gorgonian corals from the Aleutian islands. FEMS Microbiol Ecol 76:109–120. doi:10.1111/j.1574-6941.2010.01033.x. 21223327

[48] van de Water JAJM, Melkonian R, Voolstra CR, Junca H, Beraud E, Allemand D, Ferrier-Pagès C. 2017. Comparative assessment of Mediterranean Gorgonian-associated microbial communities reveals conserved core and locally variant bacteria. Microb Ecol 73:466–478. doi:10.1007/s00248-016-0858-x. 27726033

[49] Hernandez-Agreda A, Leggat W, Bongaerts P, Ainsworth TD. 2016. The microbial signature provides insight into the mechanistic basis of coral success across reef habitats. mBio 7:e00560-16. doi:10.1128/mBio.00560-16. 27460792 PMC4981706

[50] Dinsdale EA, Pantos O, Smriga S, Edwards RA, Angly F, Wegley L, Hatay M, Hall D, Brown E, Haynes M, Krause L, Sala E, Sandin SA, Thurber RV, Willis BL, Azam F, Knowlton N, Rohwer F. 2008. Microbial ecology of four coral atolls in the Northern Line Islands. PLoS One 3:e1584. doi:10.1371/journal.pone.0001584. 18301735 PMC2253183

[51] Wainwright BJ, Afiq-Rosli L, Zahn GL, Huang D. 2019. Characterisation of coral-associated bacterial communities in an urbanised marine environment shows strong divergence over small geographic scales. Coral Reefs 38:1097–1106. doi:10.1007/s00338-019-01837-1.

[52] Ding J-Y, Shiu J-H, Chen W-M, Chiang Y-R, Tang S-L. 2016. Genomic insight into the host-endosymbiont relationship of Endozoicomonas montiporae CL-33(T) with its coral host. Front Microbiol 7:251. doi:10.3389/fmicb.2016.00251. 27014194 PMC4781883

[53] Hibbing ME, Fuqua C, Parsek MR, Peterson SB. 2010. Bacterial competition: surviving and thriving in the microbial jungle. Nat Rev Microbiol 8:15–25. doi:10.1038/nrmicro2259. 19946288 PMC2879262

[54] Sheppard SK, Guttman DS, Fitzgerald JR. 2018. Population genomics of bacterial host adaptation. Nat Rev Genet 19:549–565. doi:10.1038/s41576-018-0032-z. 29973680

[55] Quigley KM, Davies SW, Kenkel CD, Willis BL, Matz MV, Bay LK. 2014. Deep-sequencing method for quantifying background abundances of symbiodinium types: exploring the rare symbiodinium biosphere in reef-building corals. PLoS One 9:e94297. doi:10.1371/journal.pone.0094297. 24728373 PMC3984134

[56] Cunning R, Silverstein RN, Baker AC. 2015. Investigating the causes and consequences of symbiont shuffling in a multi-partner reef coral symbiosis under environmental change. Proc Biol Sci 282:20141725. doi:10.1098/rspb.2014.1725. 26041354 PMC4590431

[57] Bay LK, Doyle J, Logan M, Berkelmans R. 2016. Recovery from bleaching is mediated by threshold densities of background thermo-tolerant symbiont types in a reef-building coral. R Soc Open Sci 3:160322. doi:10.1098/rsos.160322. 27429786 PMC4929921

[58] Boulotte NM, Dalton SJ, Carroll AG, Harrison PL, Putnam HM, Peplow LM, van Oppen MJ. 2016. Exploring the Symbiodinium rare biosphere provides evidence for symbiont switching in reef-building corals. ISME J 10:2693–2701. doi:10.1038/ismej.2016.54. 27093048 PMC5113844

[59] Peixoto RS, Sweet M, Villela HDM, Cardoso P, Thomas T, Voolstra CR, Høj L, Bourne DG. 2021. Coral probiotics: premise, promise, prospects. Annu Rev Anim Biosci 9:265–288. doi:10.1146/annurev-animal-090120-115444. 33321044

[60] Sheu S-Y, Lin K-R, Hsu M-Y, Sheu D-S, Tang S-L, Chen W-M. 2017. Endozoicomonas acroporae sp. nov., isolated from Acropora coral. Int J Syst Evol Microbiol 67:3791–3797. doi:10.1099/ijsem.0.002194. 28879847

[61] Tandon K, Chiang P-W, Chen W-M, Tang S-L. 2018. Draft genome sequence of Endozoicomonas acroporae strain Acr-14T, isolated from Acropora coral. Genome Announc 6:e01576-17. doi:10.1128/genomeA.01576-17. 29439049 PMC5805887

[62] Ngugi DK, Ziegler M, Duarte CM, Voolstra CR. 2020. Genomic blueprint of glycine betaine metabolism in coral metaorganisms and their contribution to reef nitrogen budgets. iScience 23:101120. doi:10.1016/j.isci.2020.101120. 32438323 PMC7240134

[63] Price-Carter M, Tingey J, Bobik TA, Roth JR. 2001. The alternative electron acceptor tetrathionate supports B12-dependent anaerobic growth of Salmonella enterica serovar typhimurium on ethanolamine or 1,2-propanediol. J Bacteriol 183:2463–2475. doi:10.1128/JB.183.8.2463-2475.2001. 11274105 PMC95162

[64] Tanod WA, Dewanto DK, Ndobe S, Riyadi PH, Putra MY. 2019. Screening of antibacterial and antioxidant activity from the soft corals Sinularia sp. and Sarcophyton sp. origin Palu Bay, Central Sulawesi, Indonesia. Squalen Bull Marine Fisheries Postharvest Biotech 14:73. doi:10.15578/squalen.v14i2.394.

[65] Dungan AM, Bulach D, Lin H, van Oppen MJH, Blackall LL. 2021. Development of a free radical scavenging bacterial consortium to mitigate oxidative stress in cnidarians. Microb Biotechnol 14:2025–2040. doi:10.1111/1751-7915.13877. 34259383 PMC8449677

[66] Wang H, Zhang Y, Bartlett DH, Xiao X. 2021. Transcriptomic analysis reveals common adaptation mechanisms under different stresses for moderately piezophilic bacteria. Microb Ecol 81:617–629. doi:10.1007/s00248-020-01609-3. 32995929

[67] Claremont M, Reid DG, Williams ST. 2011. Evolution of corallivory in the gastropod genus Drupella. Coral Reefs 30:977–990. doi:10.1007/s00338-011-0788-5.

[68] Shafir S, Gur O, Rinkevich B. 2008. A Drupella cornus outbreak in the northern Gulf of Eilat and changes in coral prey. Coral Reefs 27:379–379. doi:10.1007/s00338-008-0353-z.

[69] Nicolet KJ, Hoogenboom MO, Gardiner NM, Pratchett MS, Willis BL. 2013. The corallivorous invertebrate Drupella aids in transmission of brown band disease on the Great Barrier Reef. Coral Reefs 32:585–595. doi:10.1007/s00338-013-1010-8.

[70] Moerland MS, Scott CM, Hoeksema BW. 2016. Prey selection of corallivorous muricids at Koh Tao (Gulf of Thailand) four years after a major coral bleaching event. CTOZ 85:291–309. doi:10.1163/18759866-08503003.

[71] Nicolet KJ, Chong-Seng KM, Pratchett MS, Willis BL, Hoogenboom MO. 2018. Predation scars may influence host susceptibility to pathogens: evaluating the role of corallivores as vectors of coral disease. Sci Rep 8:5258. doi:10.1038/s41598-018-23361-y. 29588505 PMC5869713

[72] Wilson K. 2001. Preparation of genomic DNA from bacteria. Curr Protoc Mol Biol Chapter 2:Unit 2.4. doi:10.1002/0471142727.mb0204s56. 18265184

[73] Chen C-P, Tseng C-H, Chen CA, Tang S-L. 2011. The dynamics of microbial partnerships in the coral Isopora palifera. ISME J 5:728–740. doi:10.1038/ismej.2010.151. 20962876 PMC3105734

[74] Jorgensen SL, Hannisdal B, Lanzén A, Baumberger T, Flesland K, Fonseca R, Ovreås L, Steen IH, Thorseth IH, Pedersen RB, Schleper C. 2012. Correlating microbial community profiles with geochemical data in highly stratified sediments from the Arctic Mid-Ocean Ridge. Proc Natl Acad Sci USA 109:E2846–55. doi:10.1073/pnas.1207574109. 23027979 PMC3479504

[75] Edgar RC. 2013. UPARSE: highly accurate OTU sequences from microbial amplicon reads. Nat Methods 10:996–998. doi:10.1038/nmeth.2604. 23955772

[76] Schloss PD, Westcott SL, Ryabin T, Hall JR, Hartmann M, Hollister EB, Lesniewski RA, Oakley BB, Parks DH, Robinson CJ, Sahl JW, Stres B, Thallinger GG, Van Horn DJ, Weber CF. 2009. Introducing mothur: open-source, platform-independent, community-supported software for describing and comparing microbial communities. Appl Environ Microbiol 75:7537–7541. doi:10.1128/AEM.01541-09. 19801464 PMC2786419

[77] Edgar RC, Haas BJ, Clemente JC, Quince C, Knight R. 2011. UCHIME improves sensitivity and speed of chimera detection. Bioinformatics 27:2194–2200. doi:10.1093/bioinformatics/btr381. 21700674 PMC3150044

[78] Edgar RC. 2016. UNOISE2: improved error-correction for Illumina 16S and ITS amplicon sequencing. bioRxiv doi:10.1101/081257.

[79] Quast C, Pruesse E, Yilmaz P, Gerken J, Schweer T, Yarza P, Peplies J, Glöckner FO. 2013. The SILVA ribosomal RNA gene database project: improved data processing and web-based tools. Nucleic Acids Res 41:D590–D596. doi:10.1093/nar/gks1219. 23193283 PMC3531112

[80] Yilmaz P, Parfrey LW, Yarza P, Gerken J, Pruesse E, Quast C, Schweer T, Peplies J, Ludwig W, Glöckner FO. 2014. The SILVA and “all-species living tree project (LTP)” taxonomic frameworks. Nucleic Acids Res 42:D643–D648. doi:10.1093/nar/gkt1209. 24293649 PMC3965112

[81] McMurdie PJ, Holmes S. 2013. phyloseq: an R package for reproducible interactive analysis and graphics of microbiome census data. PLoS One 8:e61217. doi:10.1371/journal.pone.0061217. 23630581 PMC3632530

[82] Oksanen J, Kindt R, Legendre P, O’Hara B, Simpson GL, Solymos P. 2008. vegan: community ecology package.

[83] Wickham H. 2011. ggplot2. WIREs Comp Stat 3:180–185. doi:10.1002/wics.147.

[84] Kolde R. pheatmap: pretty heatmaps. GitHub.

[85] Cao Y. microbiomeMarker: r package for microbiome biomarker discovery. GitHub.

[86] Shiu J-H, Ding J-Y, Tseng C-H, Lou S-P, Mezaki T, Wu Y-T, Wang H-I, Tang S-L. 2018. A newly designed primer revealed high phylogenetic diversity of Endozoicomonas in coral reefs. Microbes Environ 33:172–185. doi:10.1264/jsme2.ME18054. 29760298 PMC6031392

[87] Nawrocki EP, Eddy SR. 2013. Infernal 1.1: 100-fold faster RNA homology searches. Bioinformatics 29:2933–2935. doi:10.1093/bioinformatics/btt509. 24008419 PMC3810854

[88] Kalvari I, Argasinska J, Quinones-Olvera N, Nawrocki EP, Rivas E, Eddy SR, Bateman A, Finn RD, Petrov AI. 2018. Rfam 13.0: shifting to a genome-centric resource for non-coding RNA families. Nucleic Acids Res 46:D335–D342. doi:10.1093/nar/gkx1038. 29112718 PMC5753348

[89] Trifinopoulos J, Nguyen L-T, von Haeseler A, Minh BQ. 2016. W-IQ-TREE: a fast online phylogenetic tool for maximum likelihood analysis. Nucleic Acids Res 44:W232–W235. doi:10.1093/nar/gkw256. 27084950 PMC4987875

[90] Letunic I, Bork P. 2019. Interactive Tree Of Life (iTOL) v4: recent updates and new developments. Nucleic Acids Res 47:W256–W259. doi:10.1093/nar/gkz239. 30931475 PMC6602468

[91] De Coster W, D'Hert S, Schultz DT, Cruts M, Van Broeckhoven C. 2018. NanoPack: visualizing and processing long-read sequencing data. Bioinformatics 34:2666–2669. doi:10.1093/bioinformatics/bty149. 29547981 PMC6061794

[92] Kolmogorov M, Bickhart DM, Behsaz B, Gurevich A, Rayko M, Shin SB, Kuhn K, Yuan J, Polevikov E, Smith TPL, Pevzner PA. 2020. metaFlye: scalable long-read metagenome assembly using repeat graphs. Nat Methods 17:1103–1110. doi:10.1038/s41592-020-00971-x. 33020656 PMC10699202

[93] Schubert M, Lindgreen S, Orlando L. 2016. AdapterRemoval v2: rapid adapter trimming, identification, and read merging. BMC Res Notes 9:88. doi:10.1186/s13104-016-1900-2. 26868221 PMC4751634

[94] Walker BJ, Abeel T, Shea T, Priest M, Abouelliel A, Sakthikumar S, Cuomo CA, Zeng Q, Wortman J, Young SK, Earl AM. 2014. Pilon: an integrated tool for comprehensive microbial variant detection and genome assembly improvement. PLoS One 9:e112963. doi:10.1371/journal.pone.0112963. 25409509 PMC4237348

[95] Seemann T. 2014. Prokka: rapid prokaryotic genome annotation. Bioinformatics 30:2068–2069. doi:10.1093/bioinformatics/btu153. 24642063

[96] Aziz RK, Bartels D, Best AA, DeJongh M, Disz T, Edwards RA, Formsma K, Gerdes S, Glass EM, Kubal M, Meyer F, Olsen GJ, Olson R, Osterman AL, Overbeek RA, McNeil LK, Paarmann D, Paczian T, Parrello B, Pusch GD, Reich C, Stevens R, Vassieva O, Vonstein V, Wilke A, Zagnitko O. 2008. The RAST Server: rapid annotations using subsystems technology. BMC Genomics 9:75. doi:10.1186/1471-2164-9-75. 18261238 PMC2265698

[97] Kanehisa M, Sato Y, Morishima K. 2016. BlastKOALA and GhostKOALA: KEGG tools for functional characterization of genome and metagenome sequences. J Mol Biol 428:726–731. doi:10.1016/j.jmb.2015.11.006. 26585406

[98] Couvin D, Bernheim A, Toffano-Nioche C, Touchon M, Michalik J, Néron B, Rocha EPC, Vergnaud G, Gautheret D, Pourcel C. 2018. CRISPRCasFinder, an update of CRISRFinder, includes a portable version, enhanced performance and integrates search for Cas proteins. Nucleic Acids Res 46:W246–W251. doi:10.1093/nar/gky425. 29790974 PMC6030898

[99] Yang M, Derbyshire MK, Yamashita RA, Marchler-Bauer A. 2020. NCBI’s Conserved Domain Database and tools for protein domain analysis. Curr Protoc Bioinformatics 69:e90. doi:10.1002/cpbi.90. 31851420 PMC7378889

[100] Grant JR, Stothard P. 2008. The CGView Server: a comparative genomics tool for circular genomes. Nucleic Acids Res 36:W181–W184. doi:10.1093/nar/gkn179. 18411202 PMC2447734

[101] Rodriguez-R LM, Konstantinidis KT. 2016. The enveomics collection: a toolbox for specialized analyses of microbial genomes and metagenomes. PeerJ doi:10.7287/peerj.preprints.1900v1.

