# Supplementary figures and images for: Microbiome Restructuring: Dominant Coral Bacterium Endozoicomonas Species Respond Differentially to Environmental Changes
Source: mSystems. 2022 Jun 15;7(4):e00359-22. doi: 10.1128/msystems.00359-22 (PMC9426584; doi:10.1128/msystems.00359-22)

**A**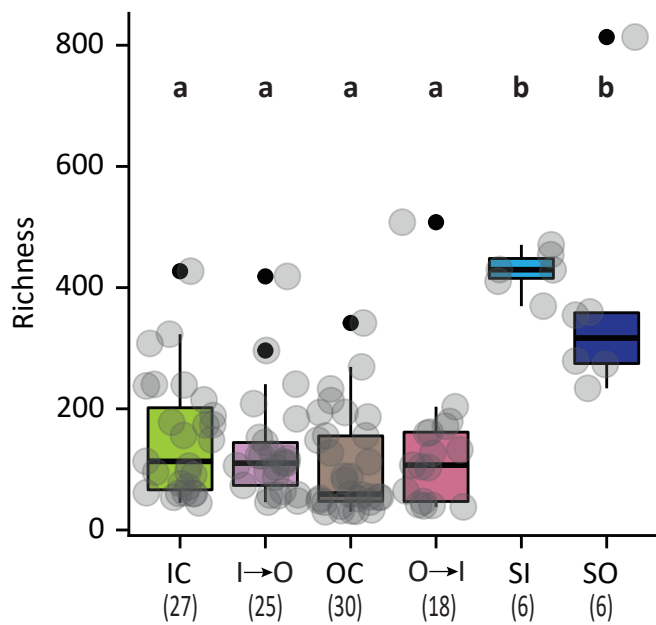**B**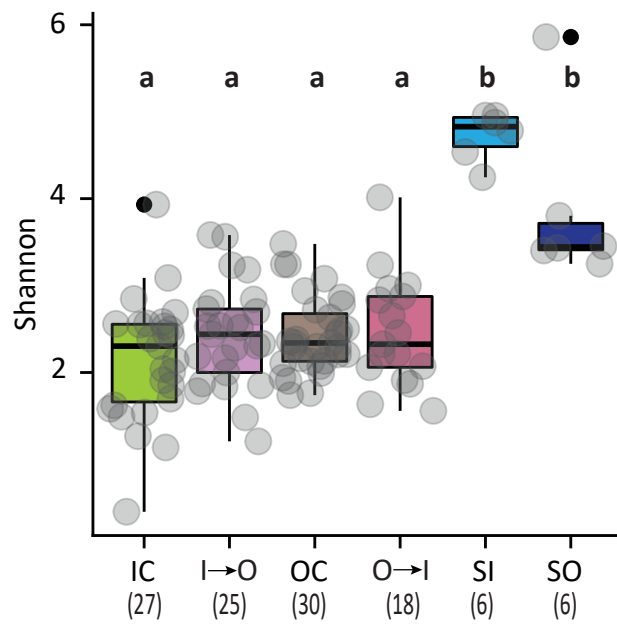**C**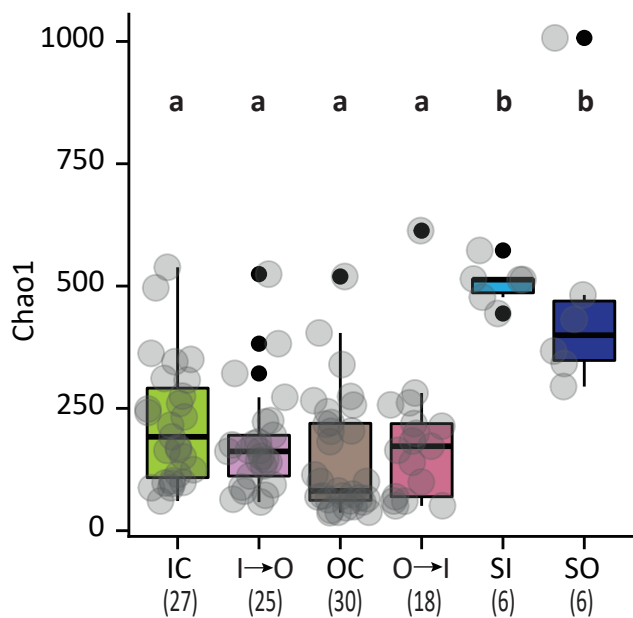**D**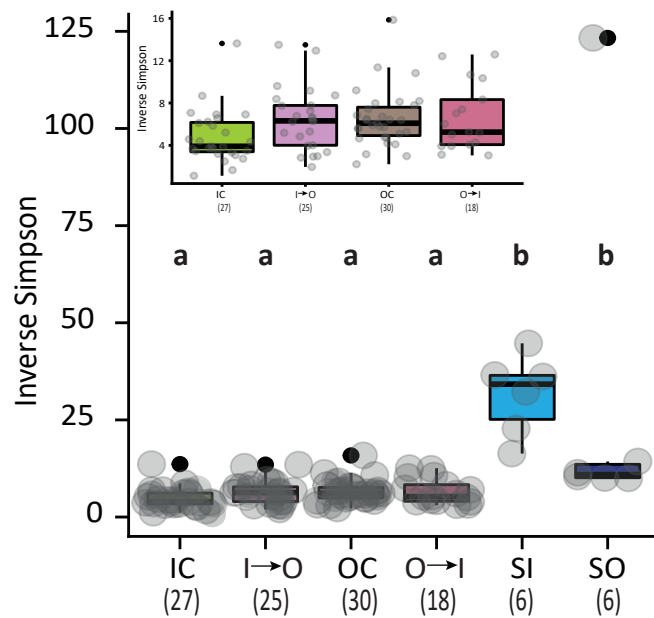

Supplement: FIG S1 [file msystems.00359-22-s0001.pdf]

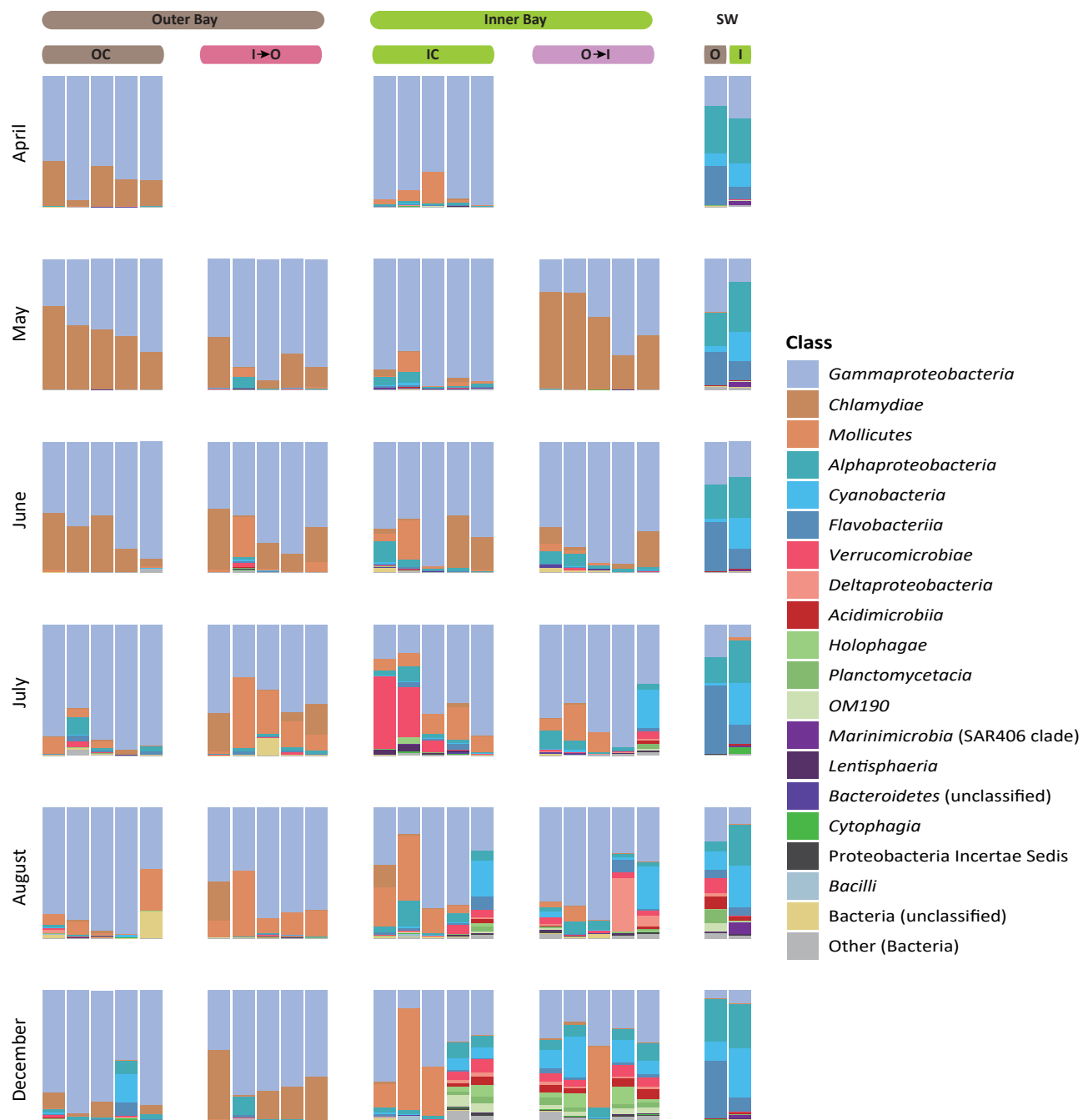

Supplement: FIG S2 [file msystems.00359-22-s0002.pdf]

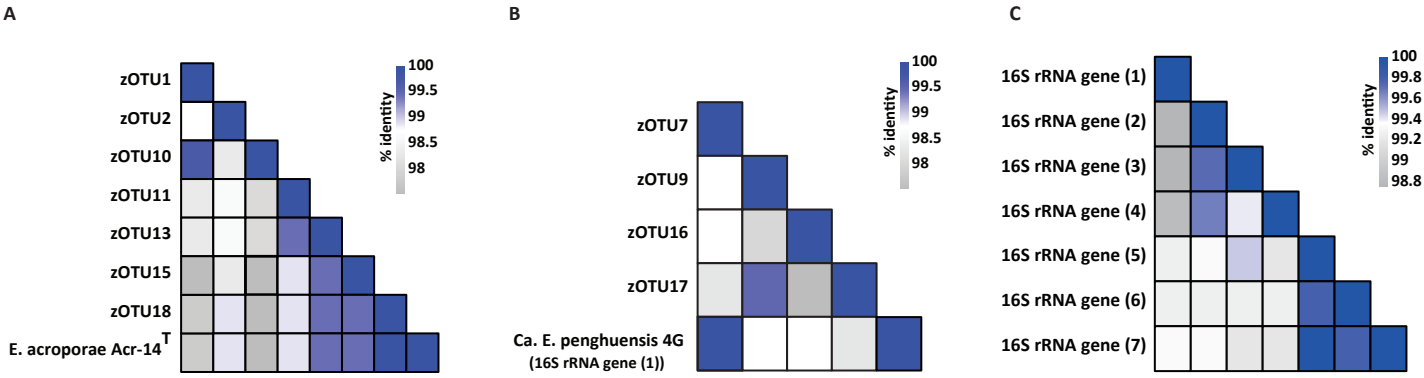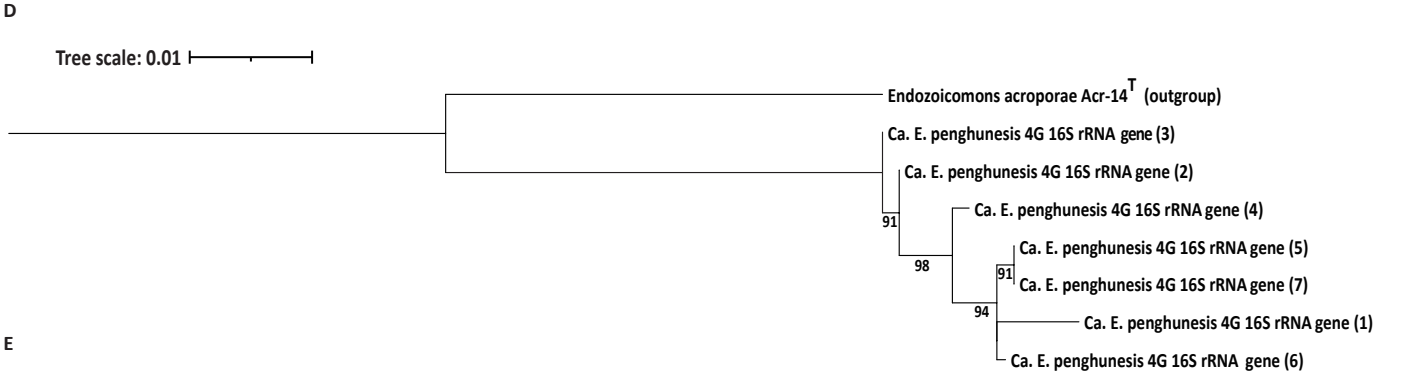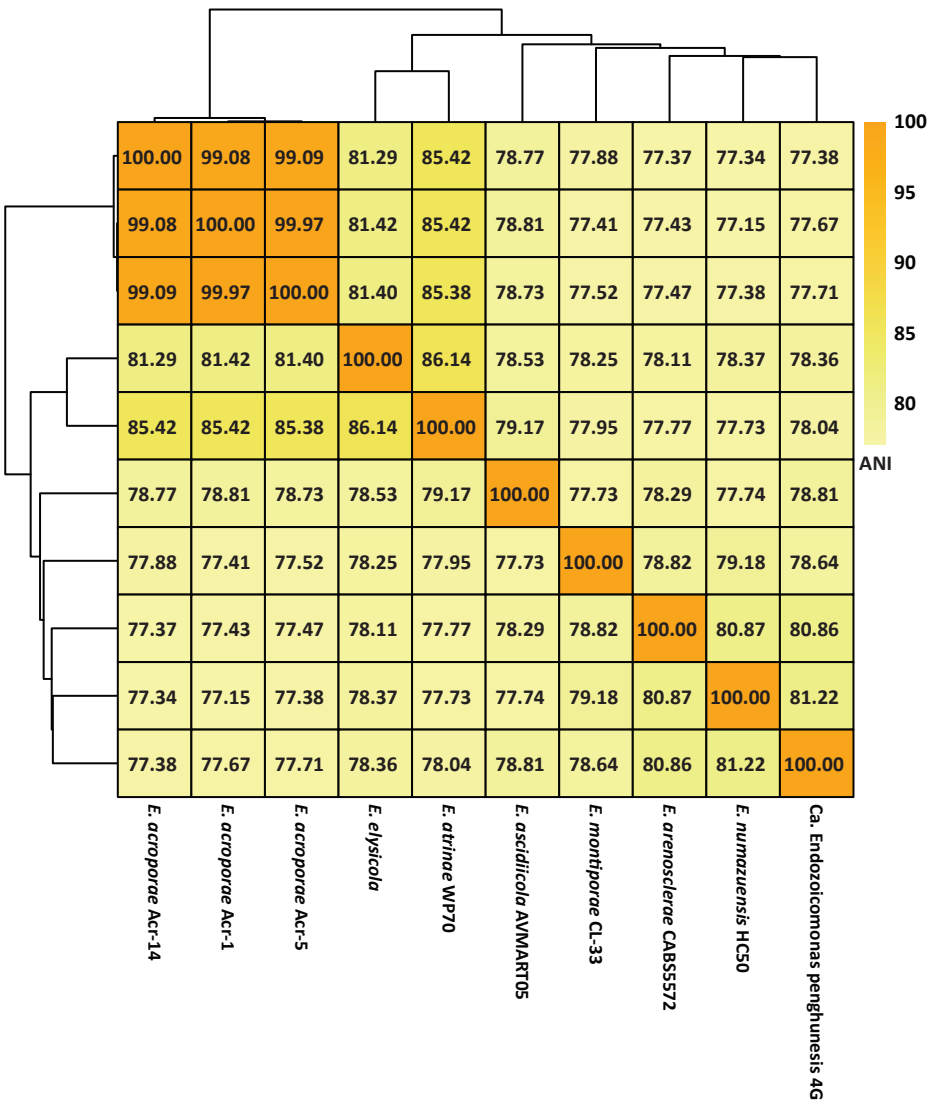

Supplement: FIG S3 [file msystems.00359-22-s0003.pdf]

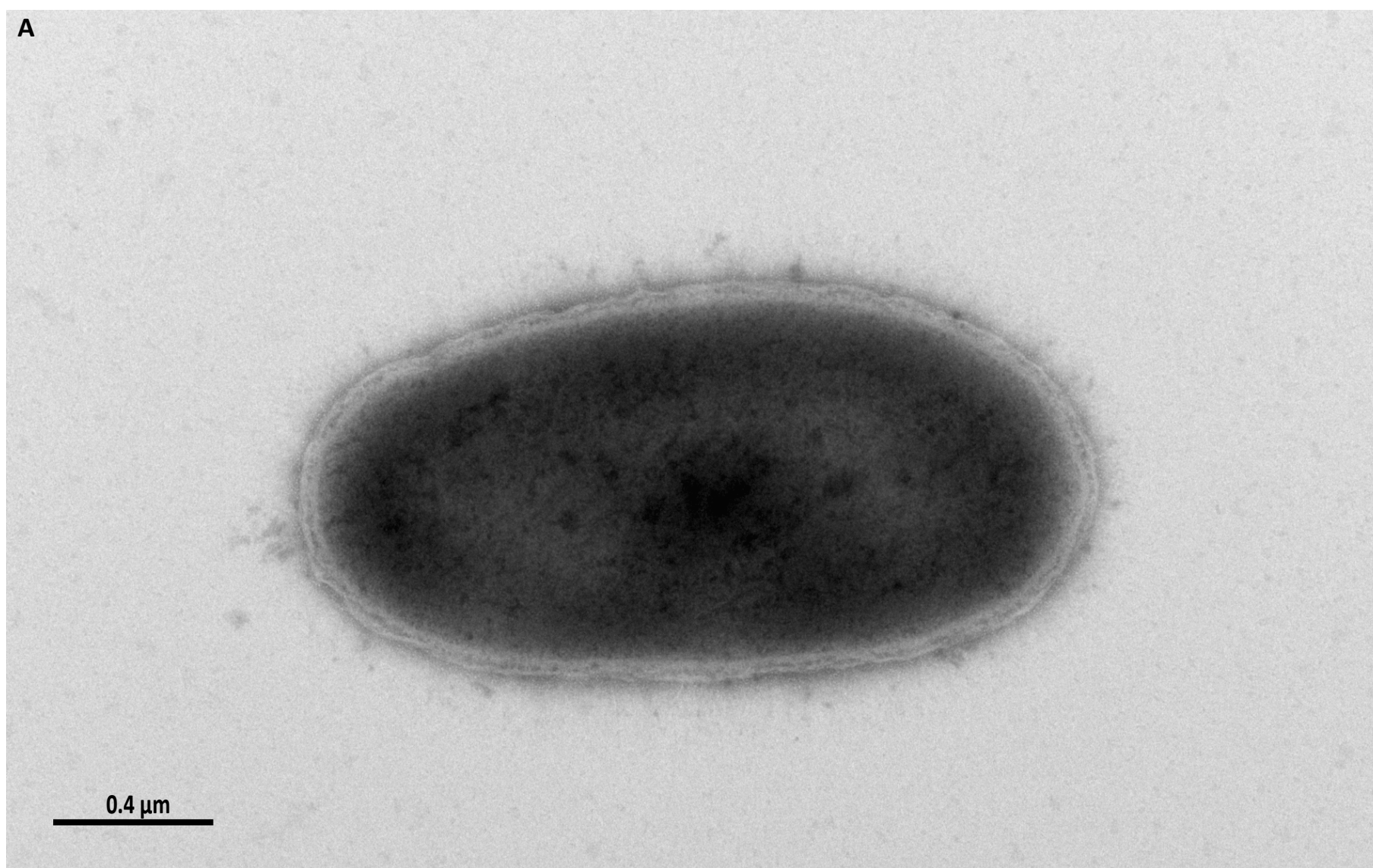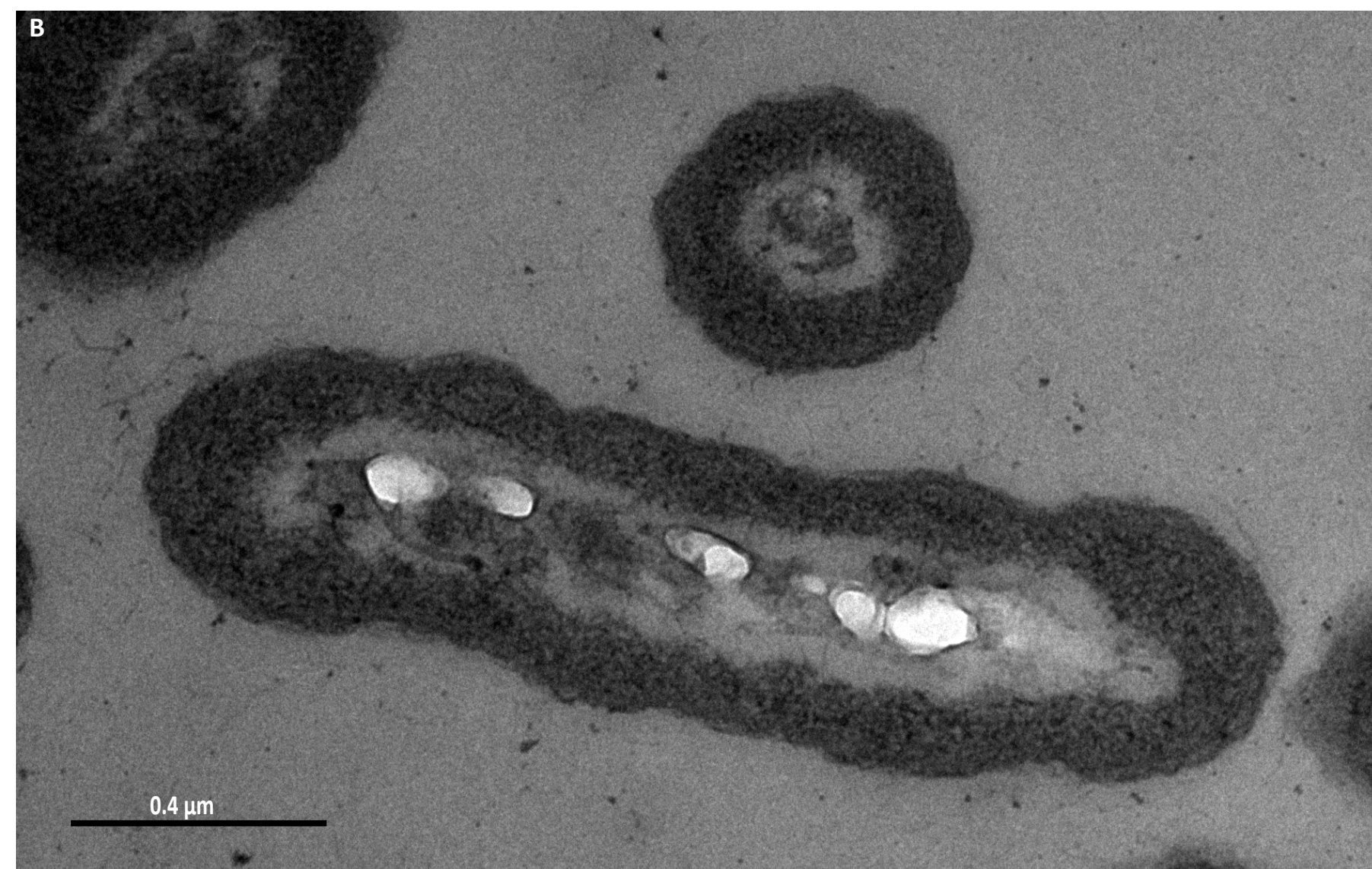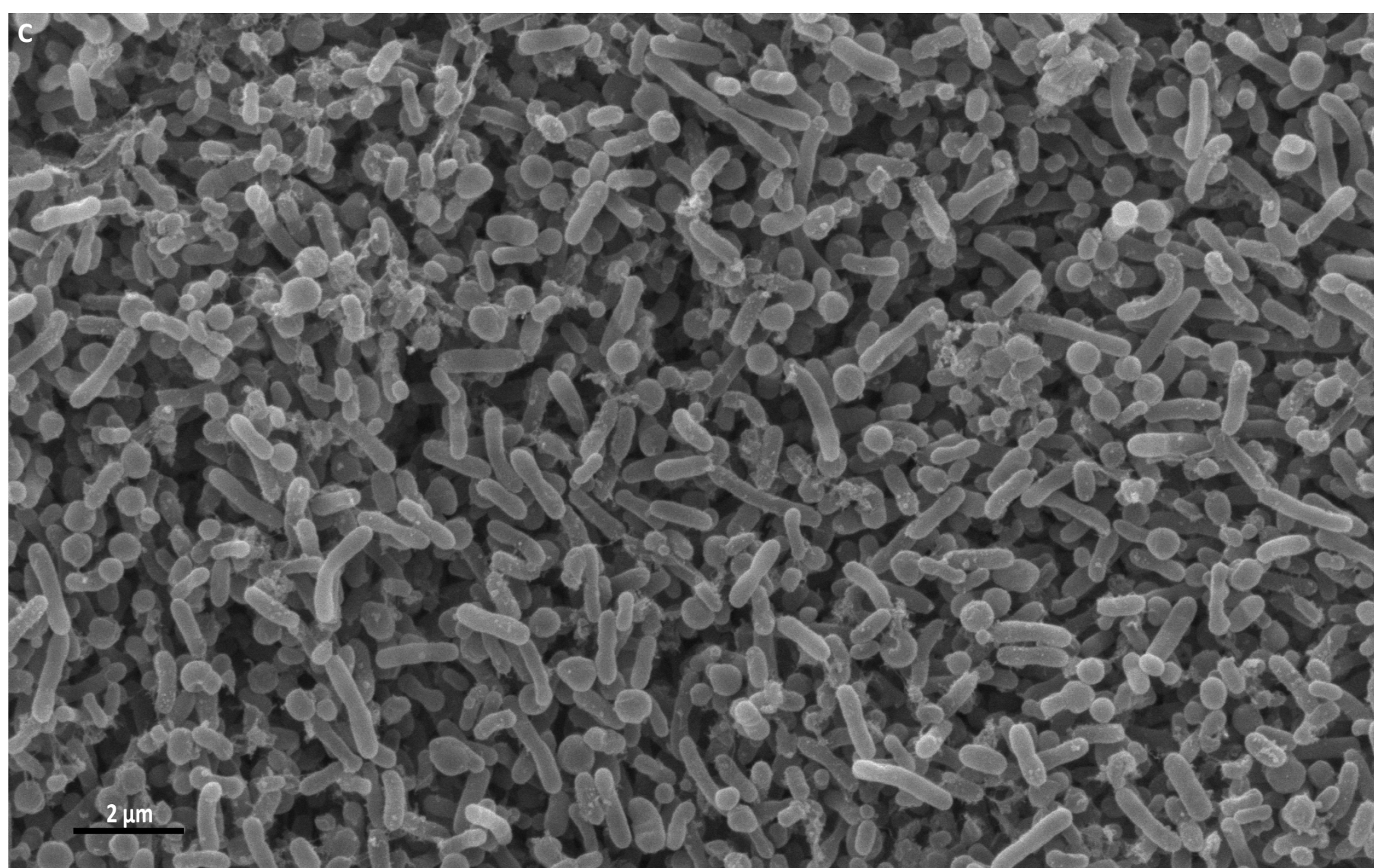

Supplement: FIG S4 [file msystems.00359-22-s0004.pdf]

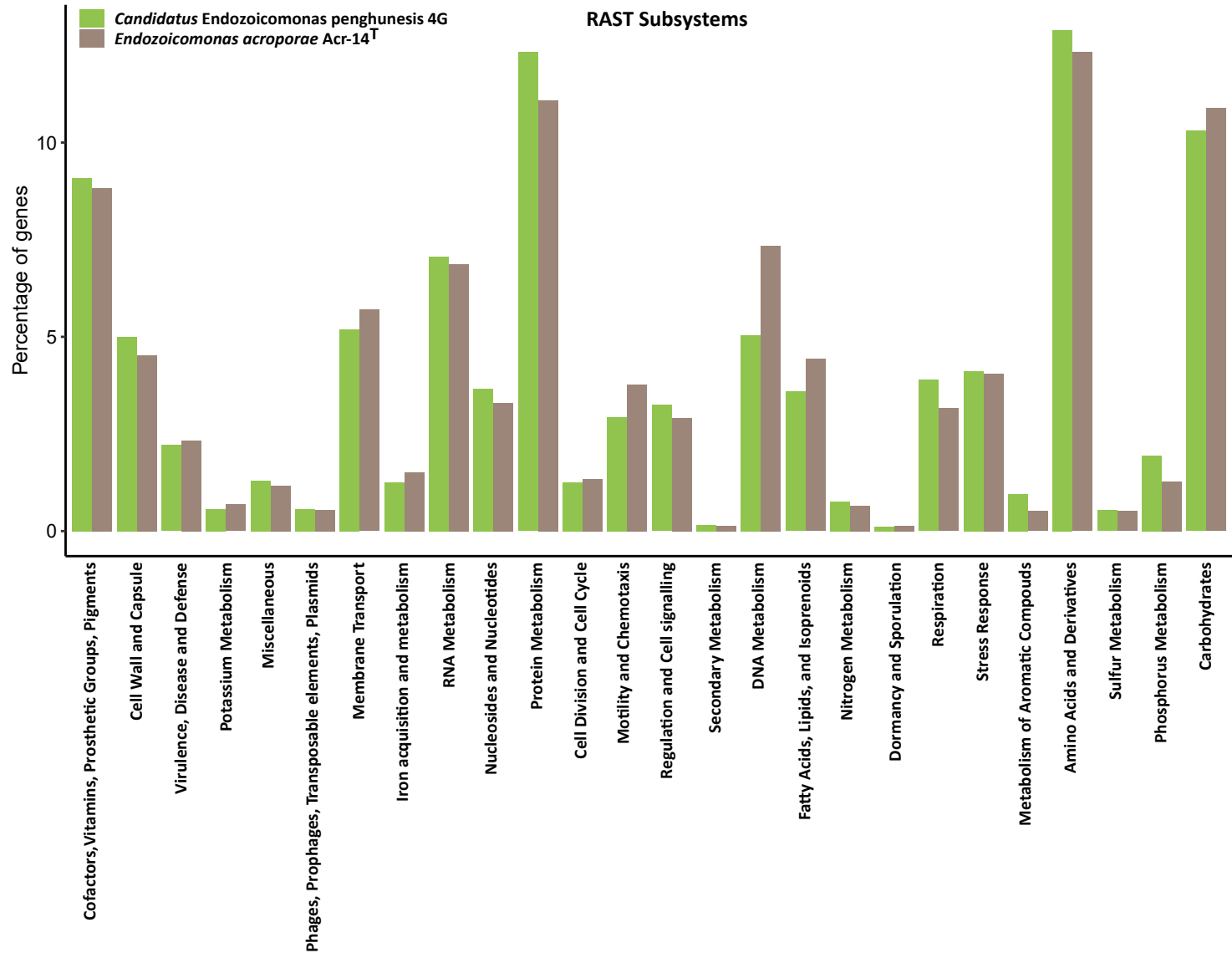

Supplement: FIG S5 [file msystems.00359-22-s0005.pdf]

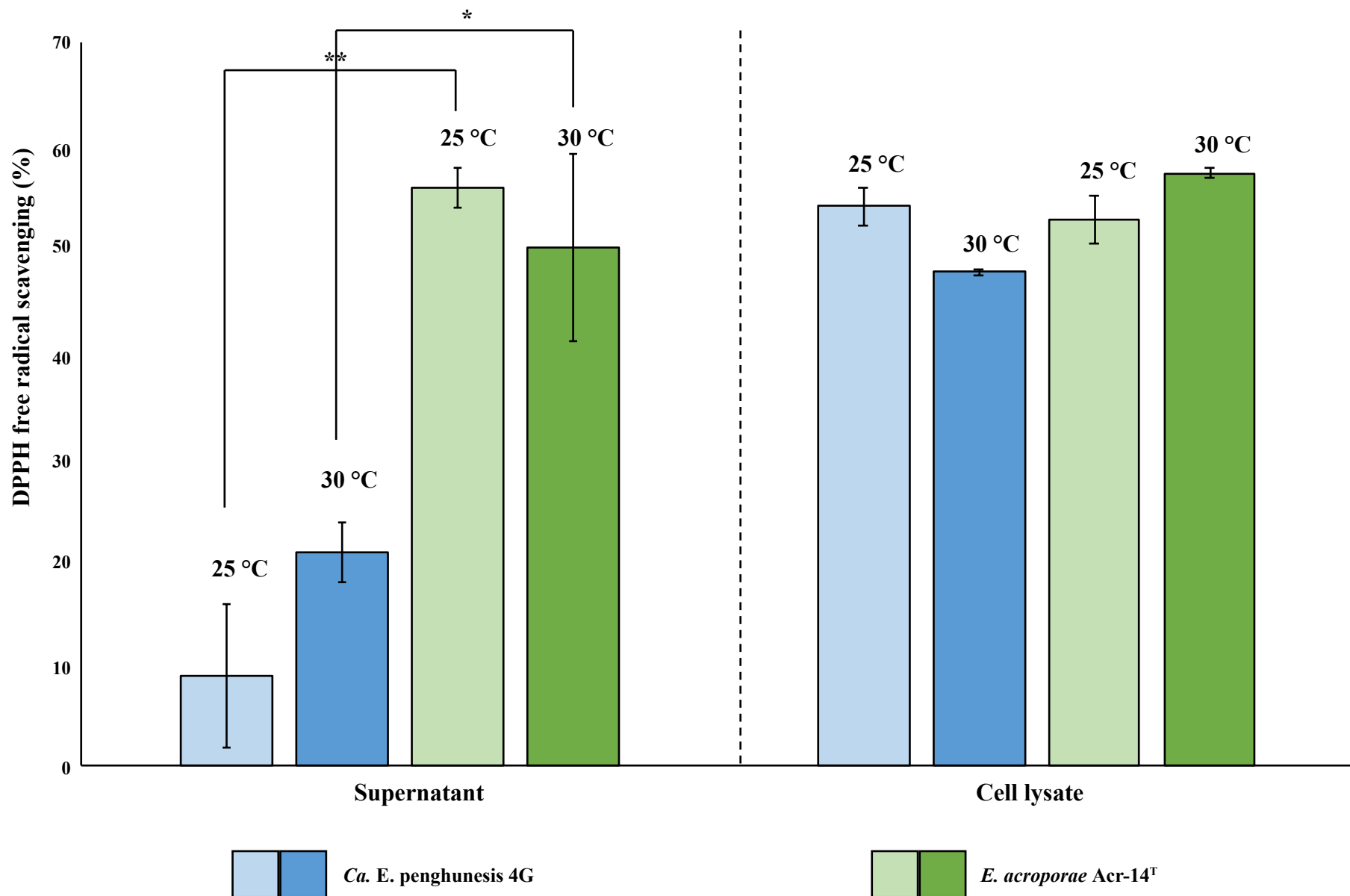

Supplement: FIG S6 [file msystems.00359-22-s0006.pdf]

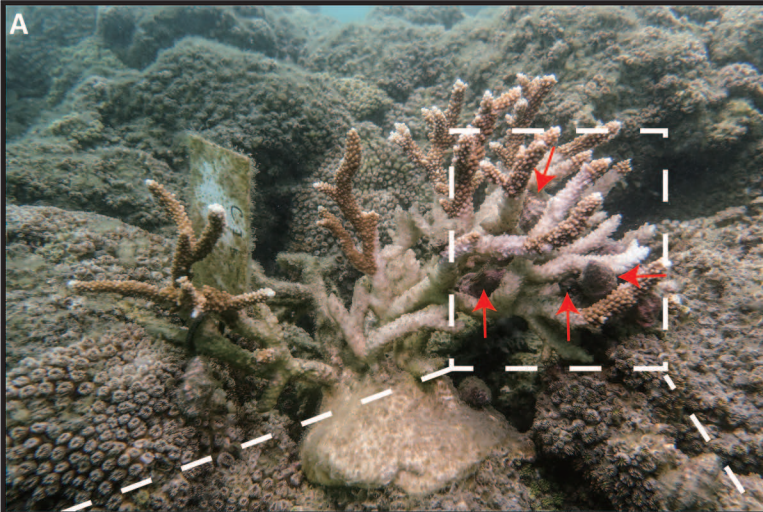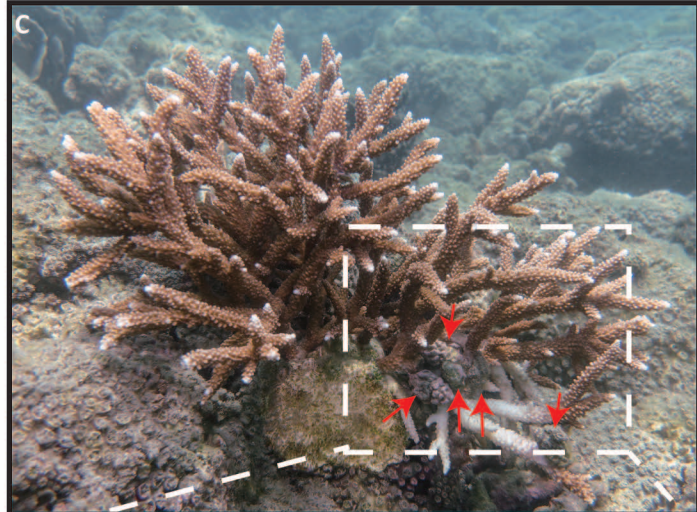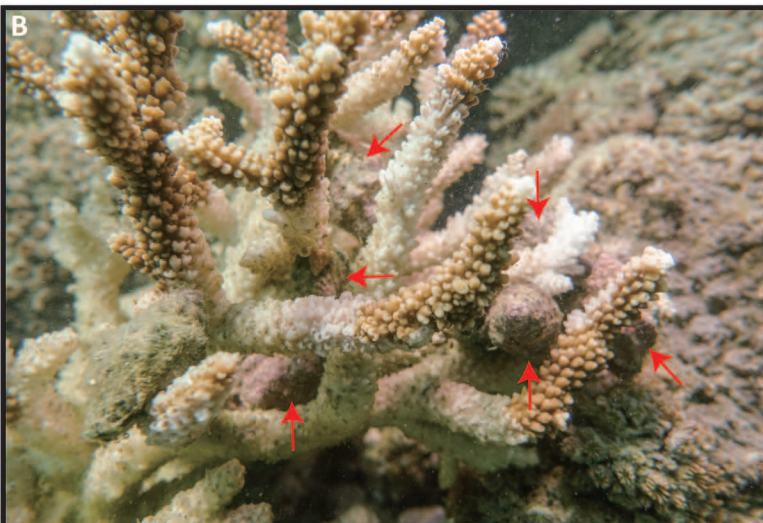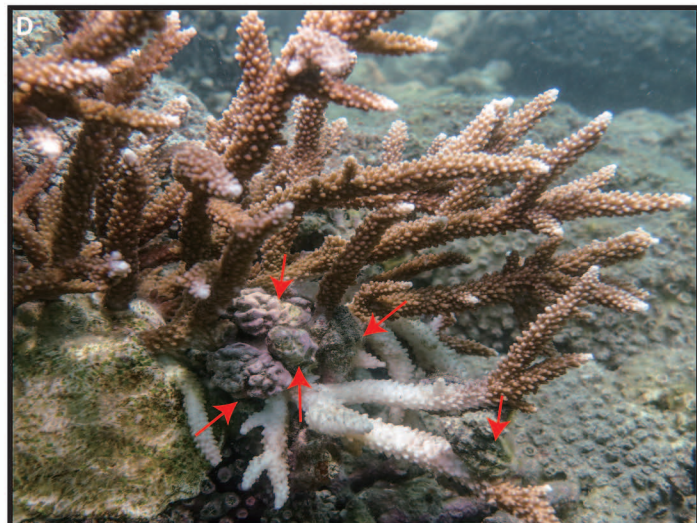

Supplement: FIG S7 [file msystems.00359-22-s0007.pdf]
